# A20 Establishes Negative Feedback With TRAF6/NF-κB and Attenuates Early Brain Injury After Experimental Subarachnoid Hemorrhage

**DOI:** 10.3389/fimmu.2021.623256

**Published:** 2021-07-26

**Authors:** Hong-Ji Deng, QuZhen Deji, WangDui Zhaba, Jia-Qiang Liu, Sheng-Qing Gao, Yan-Ling Han, Meng-Liang Zhou, Chun-Xi Wang

**Affiliations:** ^1^ Department of Neurosurgery, The First Affiliated Hospital, Guangxi Medical University, Nanning, China; ^2^ Department of Ophthalmology, Affiliated Jinling Hospital, Medical School of Nanjing University, Nanjing, China; ^3^ Department of Neurosurgery, Affiliated Jinling Hospital, Medical School of Nanjing University, Nanjing, China; ^4^ Department of Neurosurgery, Yijishan Hospital, Wannan Medical College, Wuhu, China

**Keywords:** A20, TRAF6, NF-κB, subarachnoid hemorrhage, neuroinflammation

## Abstract

Nuclear factor (NF)-κB–ty -50mediated neuroinflammation plays a crucial role in early brain injury (EBI) after subarachnoid hemorrhage (SAH). As an important negative feedback regulator of NF-κB, A20 is essential for inflammatory homeostasis. Herein, we tested the hypothesis that A20 attenuates EBI by establishing NF-κB–associated negative feedback after experimental SAH. *In vivo* and *in vitro* models of SAH were established. TPCA-1 and lentivirus were used for NF-κB inhibition and A20 silencing/overexpression, respectively. Cellular localization of A20 in the brain was determined via immunofluorescence. Western blotting and enzyme-linked immunosorbent assays were applied to observe the expression of members of the A20/tumor necrosis factor receptor-associated factor 6 (TRAF6)/NF-κB pathway and inflammatory cytokines (IL-6, IL-1β, TNF-α). Evans blue staining, TUNEL staining, Nissl staining, brain water content, and modified Garcia score were performed to evaluate the neuroprotective effect of A20. A20 expression by astrocytes, microglia, and neurons was increased at 24 h after SAH. A20 and inflammatory cytokine levels were decreased while TRAF6 expression was elevated after NF-κB inhibition. TRAF6, NF-κB, and inflammatory cytokine levels were increased after A20 silencing but suppressed with A20 overexpression. Also, Bcl-2, Bax, MMP-9, ZO-1 protein levels; Evans blue, TUNEL, and Nissl staining; brain water content; and modified Garcia score showed that A20 exerted a neuroprotective effect after SAH. A20 expression was regulated by NF-κB. In turn, increased A20 expression inhibited TRAF6 and NF-κB to reduce the subsequent inflammatory response. Our data also suggest that negative feedback regulation mechanism of the A20/TRAF6/NF-κB pathway and the neuroprotective role of A20 to attenuate EBI after SAH.

## Introduction

Aneurysmal subarachnoid hemorrhage (aSAH) is a serious disease that accounts for only 5% of all strokes but has high mortality and morbidity rates (1). Studies have shown that the overall incidence of SAH is approximately 8/100,000 persons/year with a mortality rate of up to 30% (1, 2). Two-thirds of the deaths caused by SAH occur mainly due to early brain injury (EBI) within 48 h (3). A recent study has suggested that neuroinflammation plays a key role in EBI after SAH (4). Furthermore, the NF-κB–mediated inflammatory response leads to the expression of downstream pro-inflammatory factors, causing brain damage (5).

An increasing number of studies have found that tumor necrosis factor receptor-associated factor 6 (TRAF6) is closely associated with diseases of the central nervous system, such as stroke and traumatic brain injury (6). The expression level of TRAF6 gradually increases after SAH in rats, reaching a peak level at 24 h (7). More importantly, TRAF6 acts via a ubiquitin-dependent mechanism to activate NF-κB (8). As an important regulator of inflammation, A20 is rapidly and profoundly induced by tumor necrosis factor (TNF)-α (9). Recent studies have found that A20 has dual ubiquitin editing functions to switch downstream molecule polyubiquitin chains (10). A20 is an early nuclear factor (NF)-κB responsive gene that encodes a ubiquitin-editing protein involved in the negative feedback regulation of NF-κB signaling (11). Also, the study indicated that A20 cleaves ubiquitin chains from TRAF6 to terminate NF-κB signaling (12). Although previous studies have shown that A20 exerts an important anti-inflammatory effect by inhibiting NF-κB, the specific mechanism by which A20 affects NF-κB signaling following SAH has not yet been reported.

To the best of our knowledge, few studies have systematically investigated the A20/TRAF6/NF-κB signaling pathway and the negative feedback regulation mechanism of NF-κB-induced neuroinflammation following SAH. Therefore, we aimed to verify the negative feedback mechanism of the A20/TRAF6/NF-κB pathway by interfering with NF-κB and A20 expression using *in vivo* and *in vitro* models and to explore the neuroprotective effect of A20 in inhibiting inflammatory injury.

## Materials and Methods

### Animals

All experiments involving animals were conducted following the Guide for the Care and Use of Laboratory Animals of the National Institutes of Health. All procedures were approved by the Institutional Animal Care and Use Committee. Adult C57BL/6 male mice (10–12 weeks old) weighing 20–25 g were purchased from Nanjing University (Nanjing, China). All mice were housed in a light and temperature-controlled environment (12 h light/dark cycle at 26 ± 2°C) with *ad libitum* access to food and water and adapted to the environment for 1 week before the experiment.

### Surgical Procedures

The experimental SAH model was established as previously described (13). Briefly, the mice were anesthetized with 2% isoflurane in 100% O_2_ and maintained with 1% isoflurane. An incision was made in the middle of the scalp to expose the skull, and a burr hole was drilled 4.5 mm anterior to the bregma until the dura was penetrated. A 27-gauge needle was advanced ventrally at 40° to a depth of 5 mm dorsoventral. In the sagittal plane, about 50 μl of arterial blood was injected into the pre-chiasmatic cistern for 15 s. The needle was kept in this position for 3 min to prevent cerebrospinal fluid (CSF) leakage and blood reflux. The burr hole was sealed with bone wax immediately, and the incision was surgically sutured. Mice were observed for 30 min for recovery before being returned to their cages. The injured animals were randomly assigned to drug and vehicle treatment groups for a blinded study.

### Intracerebral Ventricular Injections

Intracerebral ventricular administration was performed as previously described (13). Briefly, a Hamilton syringe was inserted into bilateral ventricles through a small cranial burr hole. According to the relative relationship with the bregma, a hole was drilled into the skull at 1.0 mm posterior, 1.5 mm lateral, and 2.5 mm deep. A total of 3 μl (1×10^8^ TU/ml) of Lv.A20-knockdown (5′-CAAAGCACUUAUUGACAGA-3′), Lv.A20-overexpression (NM_009397.3), or TPCA-1 (10 mg/kg) was injected into the lateral ventricles at a rate of 0.2 μl/min. The syringe was left *in situ* for an additional 5 min after the injection to prevent CSF leakage and reflux. The burr hole was sealed with bone wax immediately, and the incision was surgically sutured.

### Microglia Isolation

The primary microglia culture was performed as previously described (14). The cells were prepared from 1-day-old postnatal mice. The brain was isolated using Hank’s balanced salt solution (HBSS, Thermo Fisher Scientific, Waltham, MA, USA), and the cortex of the cerebral hemispheres was digested with 0.1% trypsin. The cells were seeded on a plate coated with poly-D-lysine and fed with Dulbecco’s Modified Eagle Media (DMEM, Thermo Fisher Scientific) containing 10% fetal bovine serum (FBS) and 1% penicillin-streptomycin for 2 weeks. Primary microglia cells were separated from the mixed glial cells by shaking for 2 h at 250 rpm. The cells were cultured for 1–3 days before lentivirus delivery (multiplicity of infection [MOI] = 30) and oxyhemoglobin (OxyHb, 15 µM) stimulation.

### Sample Preparation

The mice were deeply anesthetized by excess inhalation of isoflurane and perfused with 100 ml phosphate-buffered saline (PBS) at 4°C. For western blot analysis, the temporal lobe tissue was harvested on ice and used for protein extraction. The blood and culture solution were centrifuged to extract the supernatant for enzyme-linked immunosorbent assay (ELISA). For histology, mice were perfused with 100 ml PBS at 4°C followed by 100 ml of 4% paraformaldehyde. The brain was dissected and post-fixed in 4% paraformaldehyde for 24 h at 4°C. Brain hemispheres were transferred into 30% sucrose for at least 48 h and cut coronally on a microtome. Each coronary section with the temporal cortex was chosen for analysis.

### Experimental Design

**Figure 1:**
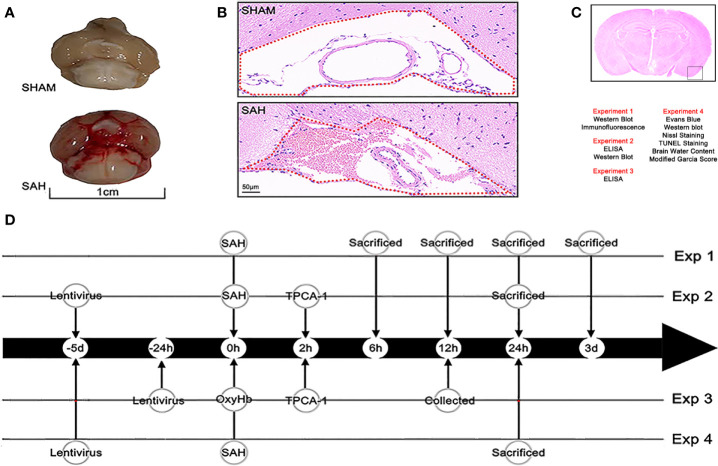
Mouse model of SAH and experimental design. **(A)** A view of the skull base brain tissue in the sham and SAH groups. Blood was deposited on the skull base in the SAH group. **(B)** Subarachnoid space of the skull base in the sham group and SAH group (hematoxylin and eosin [H&E] staining). Erythrocytes accumulated in the subarachnoid space (as shown with the red dotted line in the diagram) after injection of blood into a prechiasmatic cistern. **(C)** Observation region and the four parts of the experiment and the methods used in each part. **(D)** Flow chart of the experimental design showing the operations at different time points.

This study was completed in four experimental parts (**Figures 1A–D**). All experiment repeated three times and took the average of multiple experiments as the final experimental data.

#### Experiment 1

To detect the distribution and the expression of A20, the mice were randomly divided into the following groups: sham group and SAH groups examined at different time points (6 h, 12 h, 24 h, day 3, n = 6). The cellular location of A20 was assessed via immunofluorescence staining (n = 3). Also, the temporal lobe cortex was harvested from each group for western blotting (n = 6).

#### Experiment 2

To investigate the effect of A20 on A20/TRAF6/NF-κB signaling following SAH, the NF-κB antagonist TPCA-1 and lentivirus transfection (A20-knockdown and A20-overexpression) were used in the experiment. Mice were randomly divided into seven groups: SHAM, SAH, SAH+DMSO, SAH+Lv.GFP, SAH+TPCA-1, SAH+Lv.(A20-), and SAH+Lv.(A20+). The sham group was injected with the same volumes of normal saline as the SAH group. The SAH+DMSO group was injected with the same amount of normal DMSO as the SAH+TPCA-1 group. In the SAH+Lv.(A20-) group, lentivirus was used to knockdown A20, while in the SAH+Lv.(A20+) group, lentivirus was used to induce A20 overexpression. The SAH+Lv.GFP group was treated with an unloaded-lentivirus encoding green fluorescent protein (GFP) for comparison to the SAH+Lv.(A20-) group or SAH+Lv.(A20+) group. The temporal lobe cortex from all groups was harvested for western blotting (n = 6), and the serum samples were collected for ELISA (n = 6).

#### Experiment 3

To confirm the function of A20 in inhibiting the inflammatory response of microglia, we used TPCA-1 and lentivirus. Primary cultured microglia were randomly divided into seven groups: control, OxyHb, OxyHb+DMSO, OxyHb+Lv.GFP, OxyHb+TPCA-1, OxyHb+Lv.(A20-), and OxyHb+Lv.(A20+). The control group received no OxyHb stimulation for comparison to the OxyHb group. The OxyHb+DMSO group was treated with the same quantity of normal DMSO as the OxyHb+TPCA-1 group. The OxyHb+Lv.(A20-) and OxyHb+Lv.(A20+) groups were treated with lentivirus for A20 knockdown and overexpression, respectively. The OxyHb+Lv.GFP group was treated with unloaded-lentivirus encoding GFP for comparison to the OxyHb+Lv.(A20-) group or OxyHb+Lv.(A20+) group. Culture supernatants were collected for ELISA (n = 6).

#### Experiment 4

Several additional experimental methods were used to further investigate the neuroprotective effect of A20 in EBI after SAH. Mice were randomly assigned to five groups: SHAM, SAH, SAH+Lv.GFP, SAH+Lv.(A20-), and SAH+Lv.(A20+). The treatments were as described in experiment 2. Western blotting (n = 6), Evans blue staining (n = 6), transferase-mediated dUTP nick-end labeling (TUNEL) staining (n = 6), Nissl staining (n = 6), brain water content determination (n = 6), and modified Garcia score determination (n = 6) were performed after SAH.

### Western Blotting

Western blotting was performed as described previously (15). Briefly, each brain tissue sample was lysed in radioimmunoprecipitation assay buffer to blend 1% protease and phosphatase inhibitor cocktails. After ultrasound pyrolysis, the homogenate was centrifuged at 11,000 rpm for 15 min followed by a collection of the supernatant. The BCA kit was used to determine the total protein concentration. An equal amount of protein from each sample was loaded for separation by sodium dodecyl sulfate (SDS)-polyacrylamide gel electrophoresis (PAGE) and transferred to a polyvinylidene difluoride (PVDF) membrane for separation. The membranes were then blocked with 5% milk prepared using Tris-buffered saline containing Tween 20 (TBST) at room temperature for 2 h and then incubated with primary antibodies overnight at 4°C. The following primary antibodies were used: A20 (1:1000, ab13597, Abcam), TRAF6 (1:1000, ab62488, Abcam), IκBα (1:1000, SAB4501995, Sigma-Aldrich), p-p65 (1:1000, sc136548, Santa Cruz Biotechnology), Bax (1:1000, 14796S, Cell Signaling Technology), Bcl-2 (1:1000, ab182858, Abcam), MMP-9 (1:1000, ab38898, Abcam), ZO-1 (1:1000, 33-9100, Thermo Fisher Scientific), β-actin (1:5000, AP0060, Bioworld Technology). After the primary antibody incubation, the membranes were washed thrice for 15 min each in TBST. Afterward, the membrane was incubated in with appropriate horseradish peroxidase (HRP)-conjugated secondary antibody at room temperature for 2 h. The blot bands were visualized using enhanced chemiluminescence (ECL) and exposed to X-rays films. The protein bands were quantified using ImageJ software (NIH, Bethesda, MD, USA).

### Immunofluorescence *S*taining

Immunofluorescence staining of brain sections was performed as described previously (15). Briefly, the brain sections were fixed in 4% paraformaldehyde. After blocking with serum for 2 h, the sections were incubated at 4°C overnight with the following primary antibodies: A20 (1:100, ab13597, Abcam), NeuN (1:200, MAB377, Sigma-Aldrich), Iba-1 (1:200, MABN92, Sigma-Aldrich), GFAP (1:200, 3655S, Cell Signaling Technology). After rinsing with PBS containing 0.5% Tween-20, the sections were incubated with appropriate secondary antibodies at room temperature for 1 h. Finally, the brain sections were counterstained with 4, 6-diamidino-2-phenylindole (DAPI, 1:2000, Millipore) at room temperature for 3 min. The sections were observed under a fluorescence microscope. Six random fields in each coronary section with the temporal cortex were chosen for analysis.

### Enzyme-Linked Immunosorbent Assay

According to the manufacturer’s instructions, ELISA kits (88-7324, 88-7064, 88-7013, Thermo Fisher Scientific, *Waltham, MA, USA; ab100732, Abcam; ab253227, Abcam)* were used to evaluate the levels of inflammatory factors (interleukin IL-6, IL-1β, and TNF-α), MMP-9 and proMMP-9 in serum. Briefly, the samples were incubated with appropriate monoclonal antibodies and biotinylated anti-mice antibodies, sequentially, followed by horseradish oxidase. The measured optical density (OD) values were converted into concentration using standard curves.

### Evans Blue Dye Staining

Evans blue extravasation was used to assess the permeability of the blood–brain barrier (BBB) at 24 h after SAH. As described previously (16), Evans blue dye (2%, 4 ml/kg) was injected via a tail vein and circulated for 1 h. Mice were anesthetized and perfused with 0.9% normal saline solution to remove the circulating dye. Next, the brain specimens were removed and weighed. After ultrasound pyrolysis, an equal volume of 50% trichloroacetic acid was added to each sample for overnight incubation, followed by centrifugation at 14,000 rpm at 4°C for 30 min. The absorbance of the supernatant was measured at 620 nm using a spectrophotometer. The measured OD values were converted into concentration value using a standard curve.

### TUNEL Staining

Apoptotic cells were detected according to the manufacturer’s instructions using the apoptotic kit (Roche, South San Francisco, CA, USA). Briefly, the brain sections were deparaffinized, rehydrated, and washed with distilled water. Then the sections were incubated with TUNEL reaction mixture for 1 h at 37°C. The severity of brain injury was evaluated using the apoptotic index, which is defined as the average percentage of TUNEL-positive cells. TUNEL-positive cells in the temporal lobe cortex were observed and counted under high magnification using a double-blinded procedure. Six random fields in each coronary section with the temporal cortex were chosen, and the mean percentage of apoptotic cells in the six fields was used for the final analysis.

### Nissl *S*taining

Nissl staining was performed to evaluated normal neurons index as described previously (13). Upon staining, the cell body of damaged neurons appears shrunken with darker staining of the nuclei compared to normal neurons. The sections were dewaxed with xylene, rehydrated with alcohol, and stained with Nissl solution for 5 min. Six random fields in the temporal lobe cortex were chosen for the final analysis.

### Brain Water Content Determination

The brain water content was measured to assess the severity of brain edema as described previously (16). Briefly, mice were anesthetized and decapitated, and the brains were immediately removed and weighed to obtain wet weight values. The samples were then placed in an oven for 72 h at 80°C to obtain the dry weight. The brain water content was calculated using the formula = [(wet weight − dry weight)/wet weight] × 100%.

### Neurologic *S*coring

The neurological scores were evaluated using the modified Garcia scoring system as previously described (15). Briefly, the evaluation scores ranged from 3–18 and were derived from six tests: spontaneous activity, symmetry in the movement of four limbs, forelimbs outstretching, climbing ability, body proprioception, and the response to vibrissae stimulation. All tests were evaluated by a blinded investigator. Higher scores represent a better neurological function.

### Statistical *A*nalysis

All statistical analyses were performed using GraphPad Prism 8 (GraphPad Inc., La Jolla, CA, USA). Data are expressed as mean ± standard error of the mean (SEM). The Kolmogorov-Smirnov test was used to assess the normality of experimental data. Statistical differences were analyzed using Student’s *t*-test or one-way analysis of variance (ANOVA) with Dunnett’s test. A *p-*value < 0.05 was considered to be statistically significant.

## Results

### A20 *Expression Was Observ*ed in *Astrocytes, Mic*roglia, and *N*eurons and *I*ncreased at 24 h *A*fter SAH

**Figure 2:**
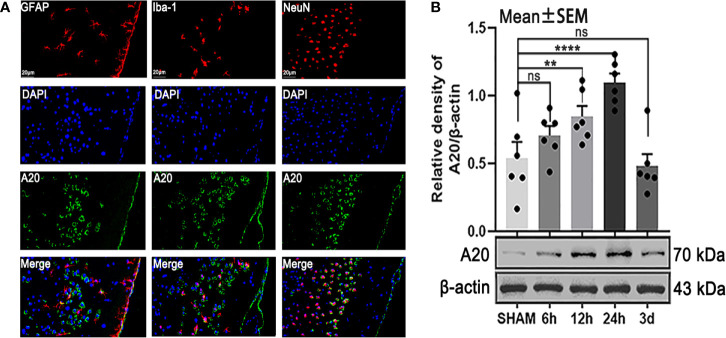
The localization and time course of A20 expression after SAH. **(A)** Immunofluorescence images showed that A20 was expressed in astrocytes, microglia, and neurons. **(B)** Western blotting showed that the peak expression of A20 appeared at 24 h after SAH. Data are expressed as mean ± SEM, n = 6, **p < 0.01, ****p < 0.001 versus sham, ns versus sham. ns, non-significant; NeuN, neuronal marker; GFAP, astrocyte marker; Iba-1, microglia marker.

To make cellular localization clear and detect changes in protein levels, we performed immunofluorescence staining and western blotting, respectively. The results of immunofluorescence staining showed that A20 was expressed in astrocytes, microglia, and neurons after SAH (**Figure 2A**) , which was consistent with previous results (17). Then, we assessed the A20 protein level at different time points following SAH. Western blot analysis demonstrated that A20 expression was significantly increased after SAH (*p* < 0.01 vs 12 h, *p* < 0.001 vs 24 h, **Figure 2B**) compared with the SHAM group. We found no significant differences between A20 expression in the SHAM group after 6 h and after 3 days. Taken together, these results suggest A20 expression in the brain is increased after SAH, and we chose the time point of 24 h after SAH for use in subsequent experiments.

### A20 Negative Feedback Regulated TRAF6 and NF-κB Expression After SAH

**Figure 3:**
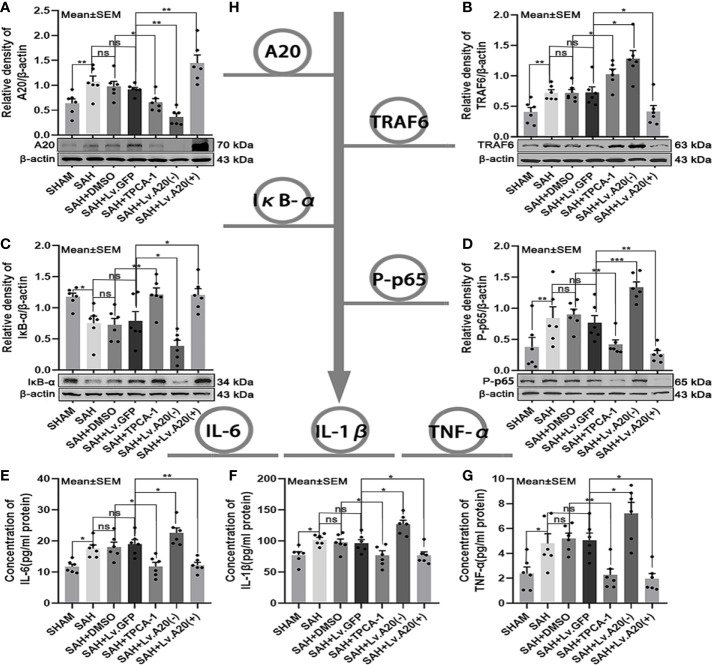
A20 negative feedback regulates the NF-κB A20/TRAF6/NF-κB signaling pathway.**(A-D)** Immunoblotting of A20, TRAF6, IκB-α, and phosphorylated (P)-p65 in brain tissue after treatment with TPCA-1 and lentivirus demonstrated a negative feedback loop between A20 and NF-κB. **(E-G)** ELISA of inflammatory cytokines—IL-6, IL-1β, and TNF-α. Administration of TPCA-1 and lentivirus knockdown inhibited the expression of the inflammatory cytokines. The administration of TPCA-1 and lentivirus to induce overexpression of A20 inhibited the release of inflammatory cytokines, and inflammatory cytokine levels were elevated by A20 knockdown. **(H)** Tree diagram of the A20/TRAF6/NF-κB signal pathway. Data are expressed as mean ± SEM, n = 6, *p < 0.05, **p < 0.01, ***p < 0.005, ns versus SAH. ns, non-significant.

We assessed the protein expression levels of the A20/TRAF6/NF-κB signaling pathway using western blotting and detected the levels of inflammatory cytokines IL-6, IL-1β, and TNF-α by ELISA (**Figure 3H**). Western blot for A20 protein expression was markedly decreased in the SHAM (*p* < 0.01) compared with the SAH group, and significantly reduced in the SAH+TPCA-1 (*p* < 0.05) compared with the SAH+DMSO group. Moreover, A20 protein was observably decreased in the SAH+Lv. (A20-) (*p* < 0.01) groups, whereas it was markedly increased in the SAH+Lv. (A20+) (*p* < 0.01) (**Figure 3A**) compared with the SAH+Lv.GFP group. Western blot for TRAF6 protein expression was inhibited in the SHAM (*p* < 0.01) compared with the SAH group, and significantly increased in the SAH+TPCA-1 (p < 0.05) compared with the SAH+DMSO group. In addition, TRAF6 protein was markedly increased in the SAH+Lv. (A20-) (*p* < 0.05) groups, whereas it was observably decreased in the SAH+Lv. (A20+) (*p* < 0.05) (**Figure 3B**) compared with the SAH+Lv.GFP group. Western blot for IκB-α protein expression was significantly improved in the SHAM (*p* < 0.01) compared with the SAH group, and significantly increased in the SAH+TPCA-1 (*p* < 0.01) compared with the SAH+DMSO group. In addition, IκB-α protein was observably reduced in the SAH+Lv. (A20-) (*p* < 0.05) groups, whereas it was markedly increased in the SAH+Lv. (A20+) (*p* < 0.05) (**Figure 3C**) compared with the SAH+Lv.GFP group. Contrary to IκB-α, P-p65 protein expression was decreased in the SHAM (*p* < 0.01) compared with the SAH group, and significantly decreased in the SAH+TPCA-1 (*p* < 0.01) compared with the SAH+DMSO group. Moreover, P-p65 protein was observably increased in the SAH+Lv. (A20-) (*p* < 0.005) groups, whereas it was observably decreased in the SAH+Lv. (A20+) (*p* < 0.01) (**Figure 3D**) compared with the SAH+Lv.GFP group. ELISA assay demonstrated that IL-6, IL-1β, and TNF-α were significantly reduced in the SHAM (*p* < 0.05) compared with the SAH group, and significantly decreased in the SAH+TPCA-1 (IL-6 and IL-1β: *p* < 0.05; TNF-α: *p* < 0.01) compared with the SAH+DMSO group. In addition, IL-6, IL-1β, and TNF-α were observably increased in the SAH+Lv. (A20-) (*p*<0.05) groups, whereas it was observably decreased in the SAH+Lv. (A20+) (IL-6: *p*<0.01; IL-1β and TNF-α: *p*<0.05) (**Figures 3E–G** **)** compared with the SAH+Lv.GFP group. We found no significant differences between the SAH, SAH+DMSO, and SAH+Lv.GFP groups. The above results indicate that a negative feedback regulation mechanism exists between A20 and the TRAF6/NF-κB signaling pathway.

### A20 *R*educed the *S*ecretion of IL-6, IL-1β, and TNF-α by *Microglia In Vitro*

**Figure 4:**
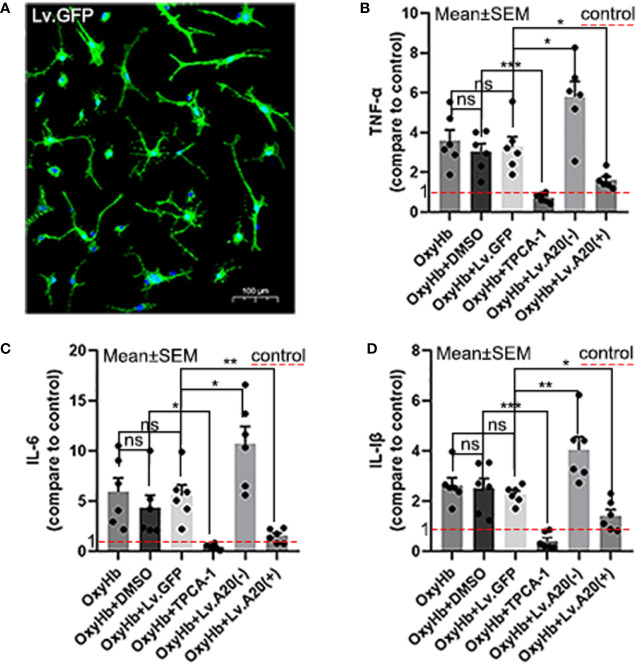
A20 influences inflammatory cytokine secretion by the microglia. **(A)** Image showing the transfection of Lv.GFP in microglia. **(B-D)** Microglia secreted inflammatory cytokines (IL-6, IL-1β, and TNF-α) after OxyHb stimulation. The red dotted line in the diagram indicated the levels of inflammatory factor secretion in the control group. Data are expressed as mean ± SEM, n = 6, *p < 0.05, **p < 0.01, ***p < 0.005, ns versus OxyHb. ns, non-significant.

We performed ELISAs to detect the expression levels of IL-6, IL-1β, and TNF-α following OxyHb stimulation *in vitro* (**Figure 4A**). The ELISA results demonstrated that IL-6, IL-1β, and TNF-α were remarkedly reduced in OxyHb+TPCA-1 (IL-6: *p*<0.05; IL-1β and TNF-α:*p*<0.001) compared with the OxyHb+DMSO group, and significantly increased in the OxyHb+Lv.(A20-) (IL-6 and TNF-α:*p*<0.05; IL-1β:*p*<0.01) while significantly decreased in OxyHb+Lv.(A20+) (IL-1β and TNF-α:*p*<0.05; IL-6: *p*<0.01) (**Figures 4B–D** **)** compared with the OxyHb+ Lv.GFP group. No significant differences were observed between the OxyHb, OxyHb+DMSO, and OxyHb+Lv.GFP groups. The above results further validate the role of A20 in the anti-inflammatory response *in vitro*.

### A20 *A*ltered MMP-9 and ZO-1 and *M*aintained BBB *Integrity After* SAH

**Figure 5:**
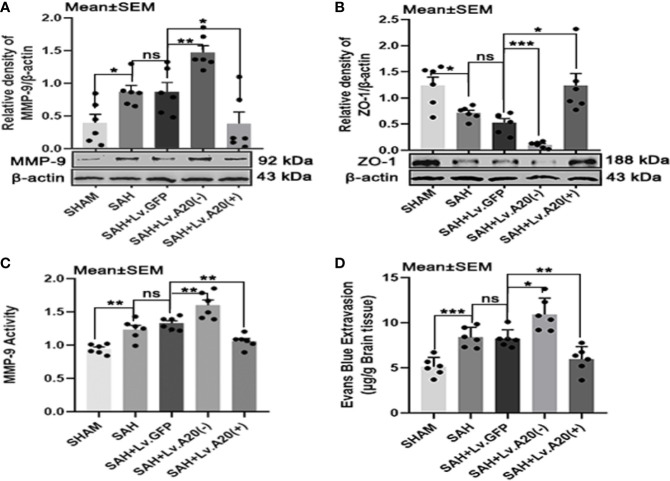
A20 affects MMP-9 and ZO-1 and maintains the integrity of the BBB after SAH. **(A, B)** Lentivirus transfection for A20 expression affected the expression of MMP-9 and ZO-1 as revealed by representative western blots. **(C)** A20 expression affected the activity of MMP-9 as revealed by ELISA. **(D)** Evaluation of the integrity of the BBB by injection of Evans blue dye. A20 played a role in maintaining the integrity of the BBB after SAH. Data are expressed as mean ± SEM, n = 6, *p < 0.05, **p < 0.01, ***p < 0.005, ns versus SAH. ns, non-significant.

To investigate the effect of A20 on MMP-9 and ZO-1 expression, we performed Western blotting to detect their protein levels after SAH. The results of Western blot analysis demonstrated that MMP-9 was significantly decreased in the SHAM (*p*<0.05) compared with the SAH group, and observably elevated in the SAH+Lv.(A20-) (*p*<0.01), while it was significantly reduced in SAH+Lv.(A20+) (*p*<0.05) (**Figure 5A**) compared with the SAH+ Lv.GFP group. On the contrary, ZO-1 protein expression was significantly increased in the SHAM (*p*<0.05) compared with the SAH group, and observably reduced in the SAH+Lv.(A20-) (*p*<0.005), while it was significantly increased in SAH+Lv.(A20+) (*p*<0.05) (**Figure 5B**) compared with the SAH+ Lv.GFP group. The results of the activity of MMP-9 showed that the activity of MMP-9 was significantly decreased in the SHAM (*p*<0.01) compared with the SAH group, and remarkedly increased in the SAH+Lv.(A20-) (*p*<0.01), while it was significantly reduced in SAH+Lv.(A20+) (*p*<0.01) (**Figure 5C**) compared with the SAH+ Lv.GFP group. Next, we determined BBB permeability using Evans blue staining in the brain. The results demonstrated that the amount of extravasated Evans blue dye was significantly decreased in the SHAM (*p*<0.005) compared with the SAH group, and remarkedly increased in the SAH+Lv.(A20-) (*p*<0.05), while it was significantly reduced in SAH+Lv.(A20+) (*p*<0.01) (**Figure 5D**) compared with the SAH+ Lv.GFP group. There were no significant differences between the SAH and SAH+Lv.GFP groups. Therefore, A20 played a role in sustaining the ZO-1 protein level, reducing the MMP-9 level and activity, and maintaining BBB integrity after SAH.

### A20 *A*ffected the *E*xpression of Bcl-2 and Bax and *D*ecreased *Neural Cell Apoptosis Aft*er SAH

**Figure 6:**
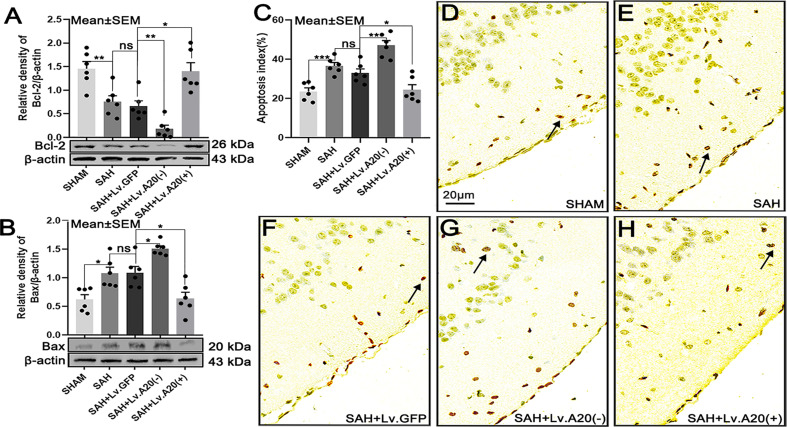
A20 influences the expression of Bcl-2 and Bax and reduces apoptosis in the cortex after SAH. **(A, B)** Immunoblotting images showing that A20 influenced Bcl-2 and Bax expression. **(C)** Bar chart of the percentages of apoptotic cells displaying that A20 reduced cortical cell apoptosis (as indicated by the black arrow). **(D–H)** TUNEL-positive cells reflect cortical apoptosis. Apoptosis decreased after using lentivirus to induce A20 overexpression. Data are expressed as mean ± SEM, n = 6, *p < 0.05, **p < 0.01, ***p < 0.005, ns versus SAH. ns, non-significant.

We detected the expression of Bcl-2 and Bax in brain tissue by western blotting after SAH. Bcl-2 protein expression was significantly increased in the SHAM (*p*<0.01), compared with the SAH group, and observably decreased in the SAH+Lv.(A20-) (*p*<0.01), while it was remarkedly increased in SAH+Lv.(A20+) (*p*<0.05) (**Figure 6A**) compared with the SAH+ Lv.GFP group. On the contrary, Bax protein expression was significantly decreased in the SHAM (*p*<0.05) compared with the SAH group, and observably increased in the SAH+Lv.(A20-) (*p*<0.05), while it was decreased in SAH+Lv.(A20+) (*p*<0.05) (**Figure 6B**) compared with the SAH+ Lv.GFP group. Then, we observed neural cell apoptosis using TUNEL staining. The results revealed that the apoptosis index was significantly decreased in the SHAM (*p*<0.005) compared with the SAH group, and observably increased in the SAH+Lv. (A20-) (*p*<0.01), while it was markedly decreased in SAH+Lv.(A20+) (*p*<0.05) (**Figures 6C–H**) compared with the SAH+ Lv.GFP group. We found no significant differences between SAH and SAH+Lv.GFP. From the above-mentioned results, we found that A20 elevated Bcl-2 expression and inhibited Bax expression as well as neural cell apoptosis in the brain.

### A20 Exerted a Protective Effect to Relieve Neuronal Injury After SAH

**Figure 7:**
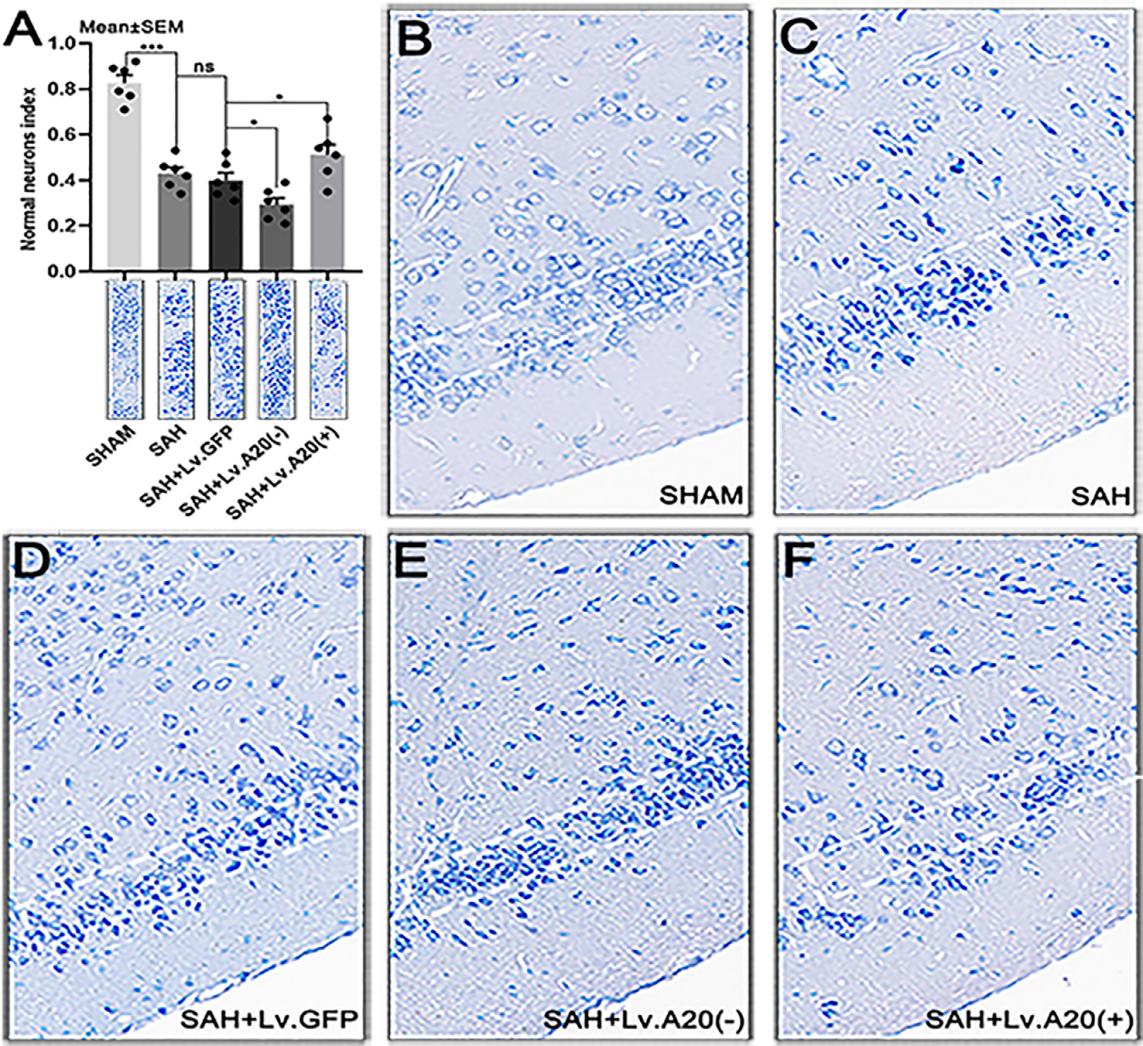
A20 promotes neuron survival in the cortex after SAH. **(A)** Bar chart of the percentages of normal neurons cells in the temporal lobe cortex. **(B–F)** Representative images of the temporal lobe cortex. The cell body of damaged neurons was shrunken, and their nuclei stained darker compared with those of normal neurons. Data are expressed as mean ± SEM, n = 6, *p < 0.05, ***p < 0.005, ns versus SAH. ns, non-significant.

We performed Nissl staining to evaluate the functional state of neurons after SAH. Most of the neurons in the sham-operated group had a large cell body and a round nucleus (**Figure 7B**), whereas damaged neurons with shrinking cell bodies, nuclear condensation, and dark cytoplasm were observed more frequently in the SAH and SAH+Lv.GFP groups (**Figures 7C, D** **)**. Furthermore, the neuronal damage was aggravated after A20 silencing and reduced after A20 upregulation (**Figures 7E, F** **)**. The result revealed that the normal neurons index was significantly increased in the SHAM (p<0.005) compared with the SAH group, and observably decreased in the SAH+Lv. (A20-) (p<0.05), while it was markedly increased in SAH+Lv.(A20+) (p<0.05) (**Figure 7A**) compared with the SAH+ Lv.GFP group. We found no significant differences between SAH and SAH+Lv.GFP. Therefore, A20 exerted a protective effect to relieve neuronal injury after SAH.

### A20 Showed a Neuroprotective Effect in Alleviating Brain Edema and Neurological Deficits After SAH

**Figure 8:**
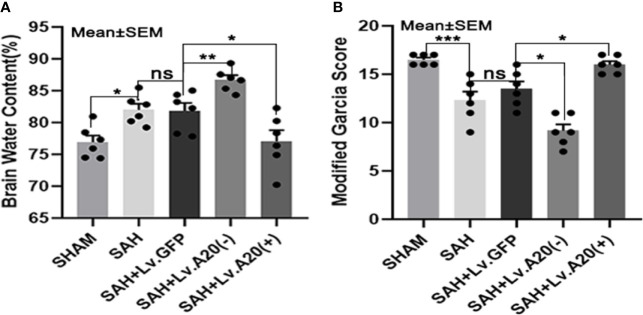
A20 attenuates brain edema and neurological deficits to exert a neuroprotective role. **(A)** Dry/wet weight ratio data represent brain water content. A20 ameliorated brain edema after SAH. **(B)** Modified Garcia score results showed that A20 remarkably improved neurological deficits in mice at 24 h after SAH. Data are expressed as mean ± SEM, n = 6, *p < 0.05, **p < 0.01, ***p < 0.005, ns versus SAH. ns, non-significant.

We evaluated brain edema and neurological deficits according to the brain water content and modified Garcia score. Based on the dry/wet weight ratio, the brain water content was dramatically reduced in the SHAM (*p*<0.05) compared with the SAH group and significantly increased in the SAH+Lv.(A20-) (*p*<0.01), while it was significantly decreased in SAH+Lv.(A20+) (*p*<0.05) (**Figure 8A**) compared with the SAH+ Lv.GFP group. The results of the modified Garcia score revealed that neurological impairment was markedly alleviated in the SHAM (*p*<0.005) compared with the SAH group and significantly aggravated in the SAH+Lv.(A20-) (*p*<0.05), while it was markedly alleviated in SAH+Lv.(A20+) (*p*<0.05) (**Figure 8B**) compared with the SAH+ Lv.GFP group. We found no significant differences between the SAH and SAH+Lv.GFP groups. Based on these results, we conclude that A20 played a neuroprotective role by alleviating brain edema and neurological impairment.

## Discussion

In this study, we hypothesized that A20 affects the negative feedback regulation of TRAF6/NF-κB, and attenuates EBI after SAH. To test this hypothesis, we established *in vivo* and *in vitro* models. The results showed that A20 was expressed in astrocytes, microglia, and neurons, and increased at 24 h after SAH. Additionally, the study had the following findings (1): the expression levels of A20 and inflammatory cytokines were suppressed while TRAF6 expression was enhanced after inhibition of NF-κB; (2) the expression levels of TRAF6, NF-κB, and inflammatory cytokines were inhibited by A20 overexpression; and (3) TRAF6, NF-κB, and inflammatory cytokine expression levels were increased after A20 knockdown. Moreover, we found that A20 played an important role in alleviating BBB damage, neural cell apoptosis, brain edema, and neurological deficits. In summary, the results of this study showed that negative feedback from A20 regulated NF-κB activity in the A20/TRAF6/NF-κB signaling pathway and exerted neuroprotective effects after SAH.

Negative feedback regulation mechanisms are widely present in physiological and pathological processes. A20 has been widely described in several inflammatory disorders (18), and impaired A20 function is associated with inflammatory diseases (19). The key is that A20 acts as a brake in the endogenous negative feedback regulation of NF-κB signaling to increase transcription rapidly after triggering of NF-κB binding sites in the A20 promoter (20). Previous research has shown that neuroinflammation plays a crucial role in brain damage after SAH (4). Therefore, we speculate that A20 expression was decreased after inhibition of NF-κB, increasing downstream expression of TRAF6 in our experiment 2. A20 serves as a brake on the inflammatory response, which leads to the suppression of TRAF6 and NF-κB expression and the downstream inflammatory response when A20 is overexpressed. Additionally, our study strongly suggests that A20 loses the ability to inhibit the subsequent expression of TRAF6, NF-κB, and inflammatory cytokines after lentivirus knockdown of A20, illustrating the function of A20 from the reverse direction. This result can be explained by previous research results: A20 is associated with TRAF6 degradation and NF-κB inhibition (21), and TRAF6 acts via a ubiquitin-dependent mechanism to activate the NF-κB pathway (8). Surprisingly, the neuroprotective effect of A20 after SAH was confirmed in our study, which suggests a promising application prospect. The above-mentioned results may explain an important mechanism for terminating inflammatory injury driven by NF-κB through the inflammatory braking effect of A20 after SAH.

Activation of NF-kB transcription factors is essential for host defense. However, after the danger is eliminated, NF-κB signaling is severely downregulated to maintain tissue homeostasis (22). A20 is an endogenous negative regulator of NF-κB signaling, which has been widely described to play roles in many inflammation-related disorders (18, 23–26). On the one hand, previous studies have demonstrated that A20 expression is elevated after traumatic brain injury (27), intracerebral hemorrhage (17, 28), and cerebral ischemia (29, 30), which is consistent with our results. On the other hand, data have increasingly found that TRAF6 is closely related to central nervous system diseases, such as stroke, traumatic brain injury, neurodegenerative diseases, and neuropathic pain (6). The expression level of TRAF6 increases gradually after SAH in rats and peaks at 24 h, which was also consistent with our results (7). Similarly, studies have shown that A20 reduces neuroinflammation and ameliorates brain injury by inhibiting the activity of TRAF6 E3 ubiquitin ligase after hemorrhagic stroke (17). Several studies provided evidence of the NF-κB inhibitory function of A20, demonstrating that A20 can prevent NF-κB activation in response to pro-inflammatory stimuli (31). As indicated previously, the silencing of A20 restores TRAF6 and NF-κB expression levels to significantly increase unchecked inflammation (21, 32, 33), whereas A20 overexpression ablates TRAF6 and dampens the inflammation cytokine production via inhibition of NF-κB activity (34). Generally, NF-κB activation is transient under continuous stimulation due to specific negative feedback regulation (11). Previous evidence showed that A20 exerts anti-inflammatory effects by directly interfering with the activation of IKKβ protein to limit NF-κB activation (30). TRAF6 acts as an E3 ubiquitin ligase to mediate IKKγ ubiquitination, leading to IκB-α degradation and p65 nuclear translocation (35). Moreover, TRAF6 inhibits autophagy and promotes oxidative stress via E3 ubiquitin ligase activity, thus exacerbating brain damage (7). Several deubiquitinating enzymes (DUBs) have been shown to negatively regulate the ubiquitin-dependent degradation mechanism of TRAF6 via deconjugation of its K63 polyubiquitin chains (8). Studies have indicated that A20 cleaves K63 polyubiquitin chains from TRAF6 to terminate NF-κB signaling (12). Studies have also shown that A20 inhibits E3 ubiquitin ligase activity of TRAF6 by antagonizing interactions with the E2 ubiquitin-conjugating enzymes, Ubc13, and UbcH5c (36). Similarly, it has been reported that A20 reduces inflammation by regulating TRAF6 polyubiquitination after intracerebral hemorrhage (17). A20 has dual ubiquitin editing functions to switch polyubiquitin chains of different configurations, which profoundly affects downstream molecules (10). The N-terminal domain of A20 is a deubiquitinating enzyme (DUB) for Lys63-linked polyubiquitinated signaling mediators, and its C-terminal domain is an ubiquitin ligase (E3) for Lys48-linked degradative polyubiquitination of the same substrate (37). Therefore, A20 can degrade TRAF6 by changing the ubiquitin-chain of TRAF6 to inhibit NF-κB as reported (21).

**Figure 9:**
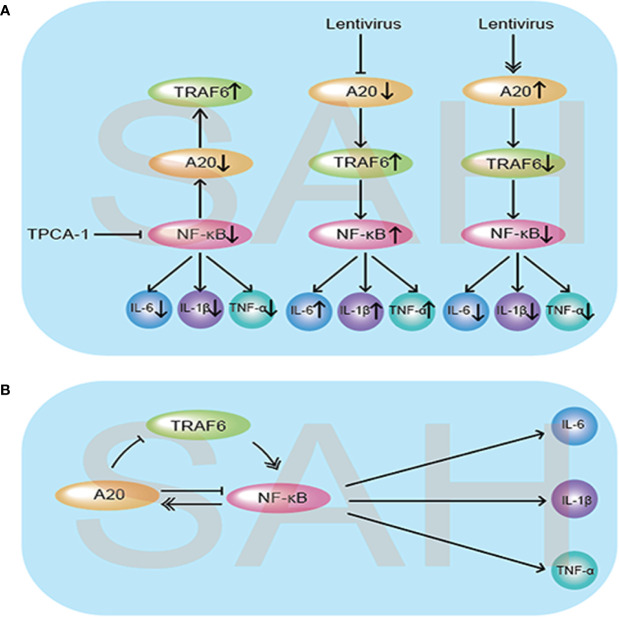
Schematic diagram illustrating the possible negative feedback loop mechanism between A20/TRAF6/NF-κB signaling after SAH. **(A)** Under the SAH conditions, inhibition of NF-κB by injection of TPCA-1 caused a reduction in inflammatory cytokine IL-6, IL-1β, and TNF-α release, and A20 expression was suppressed, which increased the expression of TRAF6. When A20 was inhibited by lentivirus, the expression levels of TRAF6 and NF-κB both increased, causing aggravation of the inflammatory response. Meanwhile, lentivirus-mediated overexpression of A20 decreased the release of inflammatory cytokines by suppressing TRAF6 and NF-κB. **(B)** As illustrated, the expression of A20 was regulated by NF-κB. In turn, the increased expression of A20 inhibited TRAF6 and NF-κB expression to reduce the subsequent inflammatory response and exert neuroprotective effects.

To the best of our knowledge, to date, there has been no study on the A20/TRAF6/NF-κB signaling pathway and the negative feedback regulation mechanism of NF-κB causing neuroinflammation after SAH. Although A20 acts as an important anti-inflammatory factor, its role has not been confirmed after SAH. By modulating the expression of NF-κB and A20, our experiments confirmed the existence of the negative feedback loop from both positive and negative perspectives after SAH (**Figures 9A, B**). Our results also suggest that A20 inhibits the expression of TRAF6, and we speculate that A20 links Lys48 polyubiquitination chains from TRAF6, leading to the degradation of TRAF6. Moreover, through lentivirus overexpression and A20 silencing, we also analyzed the effect of A20 on MMP-9 and ZO-1, thus further elucidating the effect of A20 on BBB integrity. Previous studies have shown that A20 protects cells from TNF-α–induced apoptosis (38). Therefore, we analyzed the effect of A20 on apoptosis-related proteins, Bcl-2 and Bax, further confirming the anti-apoptotic effect of A20 after SAH. Evans blue extravasation, brain edema, cell apoptosis, and neurological function score data demonstrated that A20 played an important role in alleviating EBI after SAH.

Our experiments confirmed that A20 affects the negative feedback regulation of TRAF6/NF-κB, and exerting a neuroprotective role after SAH. Furthermore, we verified the inflammatory braking effect of A20 in suppressing NF-κB endogenously. Our results provide a new therapeutic target for alleviating inflammatory damage and attenuating EBI after SAH. A20 activity is controlled by various regulatory mechanisms, including phosphorylation. Under inflammatory conditions, A20 is phosphorylated at Ser381 by IKKβ, resulting in increased activity (39). Further exploration of the role of A20 in post-translational modification, such as phosphorylation, will help us understand the anti-inflammatory function of A20 and also be conducive to the development of novel anti-inflammatory therapeutics. This study will also open new horizons for future research to explore NF-κB–mediated inflammatory mechanisms after SAH.

This study has some limitations. First, we used mice to simulate SAH in humans, but it is unclear whether the results found in mice can be translated into humans. Second, our results suggest that A20 can mitigate EBI by inhibiting NF-κB and exert anti-inflammatory effects after SAH; however, it is unclear whether A20 plays an additional role in other mechanisms after SAH.

In summary, the proposed SAH model confirms the negative feedback regulation mechanism of the A20/TRAF6/NF-κB pathway and elucidates the neuroprotective role of A20 to attenuate EBI after SAH. These findings provide a new direction in which A20 could play a critical role in reducing inflammation and provide a potential therapeutic tool for the treatment of EBI after SAH. Further studies on A20 phosphorylation are required to understand the anti-inflammatory properties of A20 in detail.

## Data Availability Statement

The original contributions presented in the study are included in the article/**Supplementary Material**. Further inquiries can be directed to the corresponding authors.

## Ethics Statement

The animal study was reviewed and approved by Institutional Animal Care and Use Committee. Written informed consent was obtained from the owners for the participation of their animals in this study.

## Author Contributions

C-XW designed the studies. H-JD carried out the surgery, physiology studies, immunohistochemical studies, and wrote the manuscript. M-LZ designed the additional experiment and contributed much to the revised manuscript. QD and WZ contributed new reagents and analytic tools. J-QL, S-QG, and Y-LH analyzed data. All authors contributed to the article and approved the submitted version.

## Funding

The study was supported by the National Natural Science Foundation, China (81601023, 81771292, and 81571162).

## Conflict of Interest

The authors declare that the research was conducted in the absence of any commercial or financial relationships that could be construed as a potential conflict of interest.

## Publisher’s Note

All claims expressed in this article are solely those of the authors and do not necessarily represent those of their affiliated organizations, or those of the publisher, the editors and the reviewers. Any product that may be evaluated in this article, or claim that may be made by its manufacturer, is not guaranteed or endorsed by the publisher.

## Abbreviations

NF-κB, nuclear factor (NF)-κB; EBI, early brain injury; SAH, subarachnoid hemorrhage; TRAF6, tumor necrosis factor receptor-associated factor 6; TUNEL, transferase-mediated dUTP nick-end labeling; Bcl-2, B-cell lymphoma (Bcl)-2; Bax, Bcl-2–associated X; MMP-9, matrix metalloproteinase-9; Zo-1, Zonula occludens-1; TNF-α, tumor necrosis factor-α; CSF, cerebrospinal fluid; ELISA, enzyme-linked immunosorbent assay; GFP, green fluorescent protein; OD, optical density; BBB, blood–brain barrier; DUB, deubiquitinating enzyme.

## References

[1] de Rooij NK Linn FH van der Plas JA Algra A Rinkel GJ. Incidence of Subarachnoid Haemorrhage: A Systematic Review With Emphasis on Region, Age, Gender and Time Trends. J Neurol Neurosurg Psychiatry (2007) 78(12):1365–72. 10.1136/jnnp.2007.117655 PMC2095631 17470467

[2] van Donkelaar CE Bakker NA Birks J Veeger N Metzemaekers JDM Molyneux AJ. Prediction of Outcome After Aneurysmal Subarachnoid Hemorrhage. Stroke (2019) 50(4):837–44. 10.1161/strokeaha.118.023902 Strokeaha118023902. 30869562

[3] Ji C Chen G. Signaling Pathway in Early Brain Injury After Subarachnoid Hemorrhage: News Update. Acta Neurochir Suppl (2016) 121:123–6. 10.1007/978-3-319-18497-5_21 26463934

[4] Lucke-Wold BP Logsdon AF Manoranjan B Turner RC McConnell E Vates GE. Aneurysmal Subarachnoid Hemorrhage and Neuroinflammation: A Comprehensive Review. Int J Mol Sci (2016) 17(4):497. 10.3390/ijms17040497 27049383 PMC4848953

[5] Sun X Ji C Hu T Wang Z Chen G. Tamoxifen as an Effective Neuroprotectant Against Early Brain Injury and Learning Deficits Induced by Subarachnoid Hemorrhage: Possible Involvement of Inflammatory Signaling. J Neuroinflamm (2013) 10:157. 10.1186/1742-2094-10-157 PMC3881500 24373431

[6] Dou Y Tian X Zhang J Wang Z Chen G. Roles of TRAF6 in Central Nervous System. Curr Neuropharmacol,4.568 (2018) 16(9):1306–13. 10.2174/1570159x16666180412094655 PMC6251041 29651950

[7] Dou Y Shen H Feng D Li H Tian X Zhang J. Tumor Necrosis Factor Receptor-Associated Factor 6 Participates in Early Brain Injury After Subarachnoid Hemorrhage in Rats Through Inhibiting Autophagy and Promoting Oxidative Stress. J Neurochem (2017) 142(3):478–92. 10.1111/jnc.14075 28543180

[8] Shi JH Sun SC. Tumor Necrosis Factor Receptor-Associated Factor Regulation of Nuclear Factor Kappab and Mitogen-Activated Protein Kinase Pathways. Front Immunol (2018) 9:1849. 10.3389/fimmu.2018.01849 30140268 PMC6094638

[9] Opipari AW Jr. Boguski MS Dixit VM. The A20 cDNA Induced by Tumor Necrosis Factor Alpha Encodes a Novel Type of Zinc Finger Protein. J Biol Chem (1990) 265(25):14705–8. 10.1016/S0021-9258(18)77165-2 2118515

[10] Chen ZJ. Ubiquitin Signalling in the NF-kappaB Pathway. Nat Cell Biol (2005) 7(8):758–65. 10.1038/ncb0805-758 PMC1551980 16056267

[11] Coornaert B Carpentier I Beyaert R. A20: Central Gatekeeper in Inflammation and Immunity. J Biol Chem (2009) 284(13):8217–21. 10.1074/jbc.R800032200 PMC2659177 19008218

[12] Boone DL Turer EE Lee EG Ahmad RC Wheeler MT Tsui C. The Ubiquitin-Modifying Enzyme A20 is Required for Termination of Toll-like Receptor Responses. Nat Immunol,23.530 (2004) 5(10):1052–60. 10.1038/ni1110 15334086

[13] Yuan B Zhou XM You ZQ Xu WD Fan JM Chen SJ. Inhibition of AIM2 Inflammasome Activation Alleviates GSDMD-induced Pyroptosis in Early Brain Injury After Subarachnoid Haemorrhage. Cell Death Dis (2020) 11(1):76. 10.1038/s41419-020-2248-z 32001670 PMC6992766

[14] Zusso M Lunardi V Franceschini D Pagetta A Lo R Stifani S. Ciprofloxacin and Levofloxacin Attenuate Microglia Inflammatory Response Via TLR4/NF-kB Pathway. J Neuroinflamm (2019) 16(1):148. 10.1186/s12974-019-1538-9 PMC6637517 31319868

[15] Gao SQ Liu JQ Han YL Deji QZ Zhaba WD Deng HJ. Neuroprotective Role of Glutathione Peroxidase 4 in Experimental Subarachnoid Hemorrhage Models. Life Sci,3.448 (2020) 257:118050. 10.1016/j.lfs.2020.118050 32634425

[16] Wang CX Xie GB Zhou CH Zhang XS Li T Xu JG. Baincalein Alleviates Early Brain Injury After Experimental Subarachnoid Hemorrhage in Rats: Possible Involvement of TLR4/NF-kappaB-mediated Inflammatory Pathway. Brain Res (2015) 1594:245–55. 10.1016/j.brainres.2014.10.014 25451085

[17] Meng Z Zhao T Zhou K Zhong Q Wang Y Xiong X. A20 Ameliorates Intracerebral Hemorrhage-Induced Inflammatory Injury by Regulating Traf6 Polyubiquitination. J Immunol (2017) 198(2):820–31. 10.4049/jimmunol.1600334 PMC5220121 27986908

[18] Momtazi G Lambrecht BN Naranjo JR Schock BC. Regulators of A20 (TNFAIP3): New Drug-Able Targets in Inflammation. Am J Physiol Lung Cell Mol Physiol (2019) 316(3):L456–l469. 10.1152/ajplung.00335.2018 30543305

[19] Polykratis A Martens A Eren RO Shirasaki Y Yamagishi M Yamaguchi Y. A20 Prevents Inflammasome-Dependent Arthritis by Inhibiting Macrophage Necroptosis Through its ZnF7 Ubiquitin-Binding Domain. Nat Cell Biol (2019) 21(6):731–42. 10.1038/s41556-019-0324-3 31086261

[20] Krikos A Laherty CD Dixit VM. Transcriptional Activation of the Tumor Necrosis Factor Alpha-Inducible Zinc Finger Protein, A20, is Mediated by Kappa B Elements. J Biol Chem (1992) 267(25):17971–6. 10.1016/S0021-9258(19)37138-8 1381359

[21] Mabilleau G Chappard D Sabokbar A. Role of the A20-TRAF6 Axis in Lipopolysaccharide-Mediated Osteoclastogenesis. J Biol Chem (2011) 286(5):3242–9. 10.1074/jbc.M110.150300 PMC3030329 21127049

[22] Ruland J. Return to Homeostasis: Downregulation of NF-κb Responses. Nat Immunol,23.530 (2011) 12(8):709–14. 10.1038/ni.2055 21772279

[23] Li KZ Liao ZY Li YX Ming ZY Zhong JH Wu GB. A20 Rescues Hepatocytes From Apoptosis Through the NF-kappaB Signaling Pathway in Rats With Acute Liver Failure. Biosci Rep,2.535 (2019) 39(1):1–13. 10.1042/bsr20180316 PMC6328859 30446523

[24] Voet S Mc Guire C Hagemeyer N Martens A Schroeder A Wieghofer P. A20 Critically Controls Microglia Activation and Inhibits Inflammasome-Dependent Neuroinflammation. Nat Commun (2018) 9(1):2036. 10.1038/s41467-018-04376-5 29789522 PMC5964249

[25] Wertz IE Newton K Seshasayee D Kusam S Lam C Zhang J. Phosphorylation and Linear Ubiquitin Direct A20 Inhibition of Inflammation. Nature (2015) 528(7582):370–5. 10.1038/nature16165 26649818

[26] Guedes RP Csizmadia E Moll HP Ma A Ferran C da Silva CG. A20 Deficiency Causes Spontaneous Neuroinflammation in Mice. J Neuroinflamm (2014) 11:122. 10.1186/1742-2094-11-122 PMC4128606 25026958

[27] Bao Z Fan L Zhao L Xu X Liu Y Chao H. Silencing of A20 Aggravates Neuronal Death and Inflammation After Traumatic Brain Injury: A Potential Trigger of Necroptosis. Front Mol Neurosci,3.720 (2019) 12:222. 10.3389/fnmol.2019.00222 PMC6761256 31607859

[28] Lu J Sun Z Fang Y Zheng J Xu S Xu W. Melatonin Suppresses Microglial Necroptosis by Regulating Deubiquitinating Enzyme A20 After Intracerebral Hemorrhage. Front Immunol (2019) 10:1360. 10.3389/fimmu.2019.01360 31258534 PMC6587666

[29] Zhang R Xu L Zhang D Hu B Luo Q Han D. Cardioprotection of Ginkgolide B on Myocardial Ischemia/Reperfusion-Induced Inflammatory Injury Via Regulation of A20-NF-kappaB Pathway. Front Immunol (2018) 9:2844. 10.3389/fimmu.2018.02844 30619251 PMC6299132

[30] Zhan J Qin W Zhang Y Jiang J Ma H Li Q. Upregulation of Neuronal Zinc Finger Protein A20 Expression is Required for Electroacupuncture to Attenuate the Cerebral Inflammatory Injury Mediated by the Nuclear Factor-Kb Signaling Pathway in Cerebral Ischemia/Reperfusion Rats. J Neuroinflamm (2016) 13(1):258. 10.1186/s12974-016-0731-3 PMC5048665 27716383

[31] Beyaert R Heyninck K Van Huffel S. A20 and A20-binding Proteins as Cellular Inhibitors of Nuclear Factor-Kappa B-dependent Gene Expression and Apoptosis. Biochem Pharmacol (2000) 60(8):1143–51. 10.1016/s0006-2952(00)00404-4 11007952

[32] Wertz IE Dixit VM. Signaling to NF-kappaB: Regulation by Ubiquitination. Cold Spring Harb Perspect Biol,9.110 (2010) 2(3):a003350. 10.1101/cshperspect.a003350 PMC2829959 20300215

[33] Xiong Y Qiu F Piao W Song C Wahl LM Medvedev AE. Endotoxin Tolerance Impairs IL-1 Receptor-Associated Kinase (IRAK) 4 and TGF-beta-activated Kinase 1 Activation, K63-linked Polyubiquitination and Assembly of IRAK1, TNF Receptor-Associated Factor 6, and IkappaB Kinase Gamma and Increases A20 Expression. J Biol Chem (2011) 286(10):7905–16. 10.1074/jbc.M110.182873 PMC3048677 21220427

[34] Li Y Mooney EC Holden SE Xia XJ Cohen DJ Walsh SW. A20 Orchestrates Inflammatory Response in the Oral Mucosa Through Restraining NF-Kappab Activity. J Immunol (2019) 202(7):2044–56. 10.4049/jimmunol.1801286 PMC6420508 30760622

[35] Chen ZJ. Ubiquitination in Signaling to and Activation of IKK. Immunol Rev,11.292 (2012) 246(1):95–106. 10.1111/j.1600-065X.2012.01108.x PMC3549672 22435549

[36] Shembade N Ma A Harhaj EW. Inhibition of NF-kappaB Signaling by A20 Through Disruption of Ubiquitin Enzyme Complexes. Science (2010) 327(5969):1135–9. 10.1126/science.1182364 PMC3025292 20185725

[37] Lin SC Chung JY Lamothe B Rajashankar K Lu M Lo YC. Molecular Basis for the Unique Deubiquitinating Activity of the NF-kappaB Inhibitor A20. J Mol Biol,5.067 (2008) 376(2):526–40. 10.1016/j.jmb.2007.11.092 PMC2346432 18164316

[38] Priem D Devos M Druwe S Martens A Slowicka K Ting AT. A20 Protects Cells From TNF-induced Apoptosis Through Linear Ubiquitin-Dependent and -Independent Mechanisms. Cell Death Dis (2019) 10(10):692. 10.1038/s41419-019-1937-y 31534131 PMC6751190

[39] Martens A van Loo G. A20 Phosphorylation Controls A20 Function. Nat Immunol,23.530 (2019) 20(10):1261–2. 10.1038/s41590-019-0481-3 31534239

