# Supplementary figures and images for: A20 Establishes Negative Feedback With TRAF6/NF-κB and Attenuates Early Brain Injury After Experimental Subarachnoid Hemorrhage
Source: Front Immunol. 2021 Jul 26;12:623256. doi: 10.3389/fimmu.2021.623256 (PMC8350325; doi:10.3389/fimmu.2021.623256)

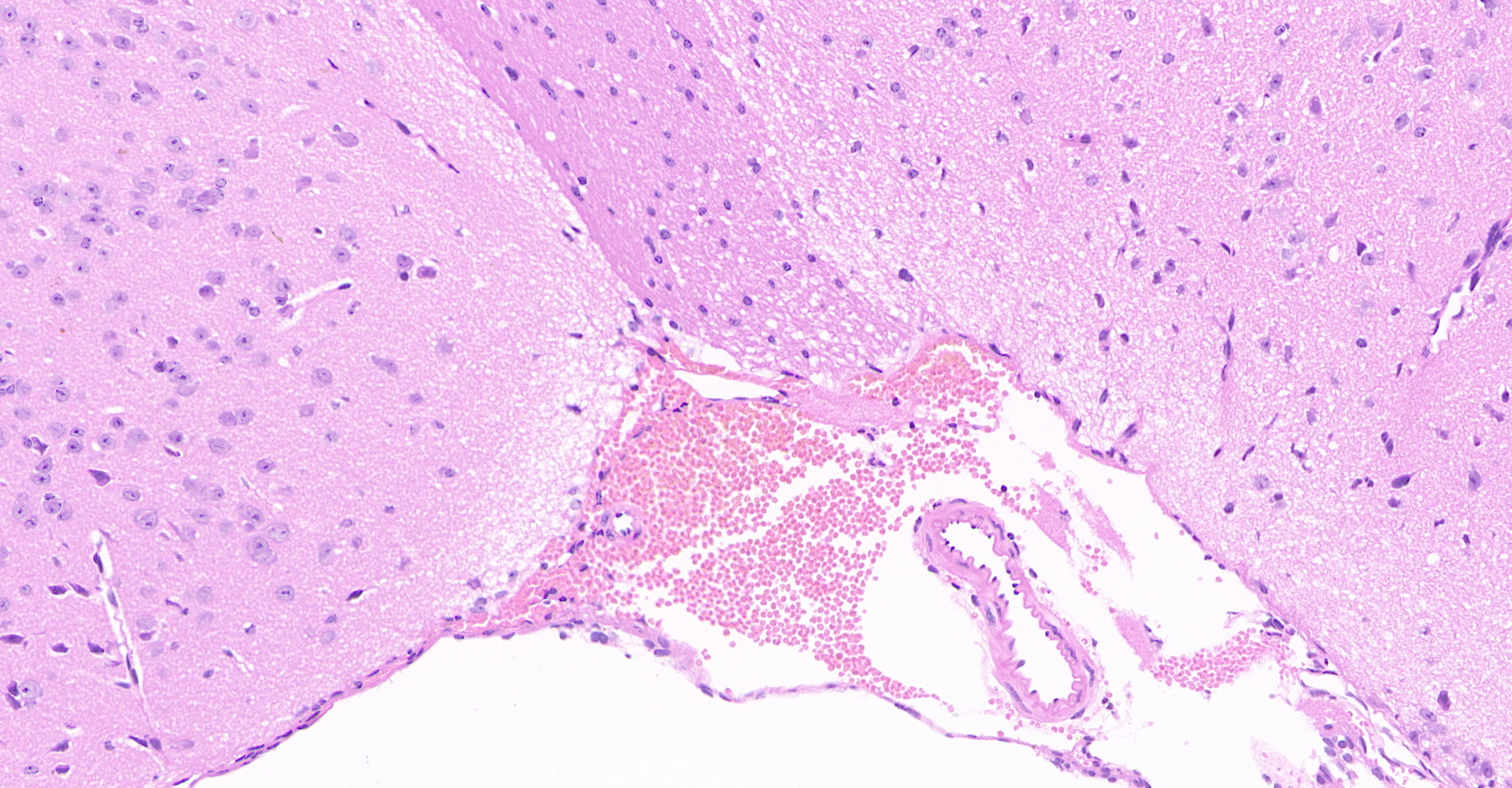

Supplement: Supplementary file 1 [file DataSheet_1.zip › Original Images/Figure 1B SAH.jpg]

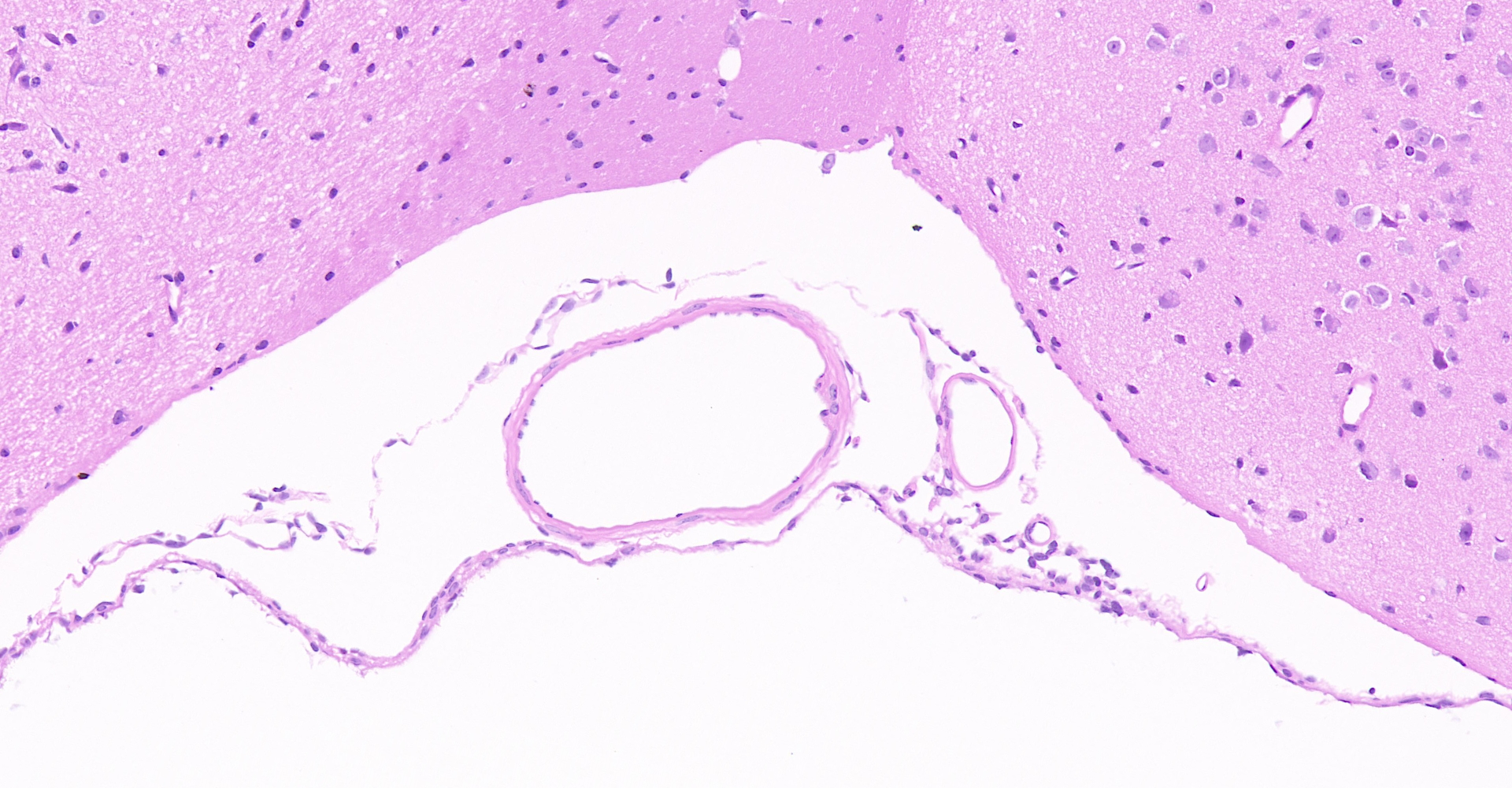

Supplement: Supplementary file 1 [file DataSheet_1.zip › Original Images/Figure 1B SHAM.jpg]

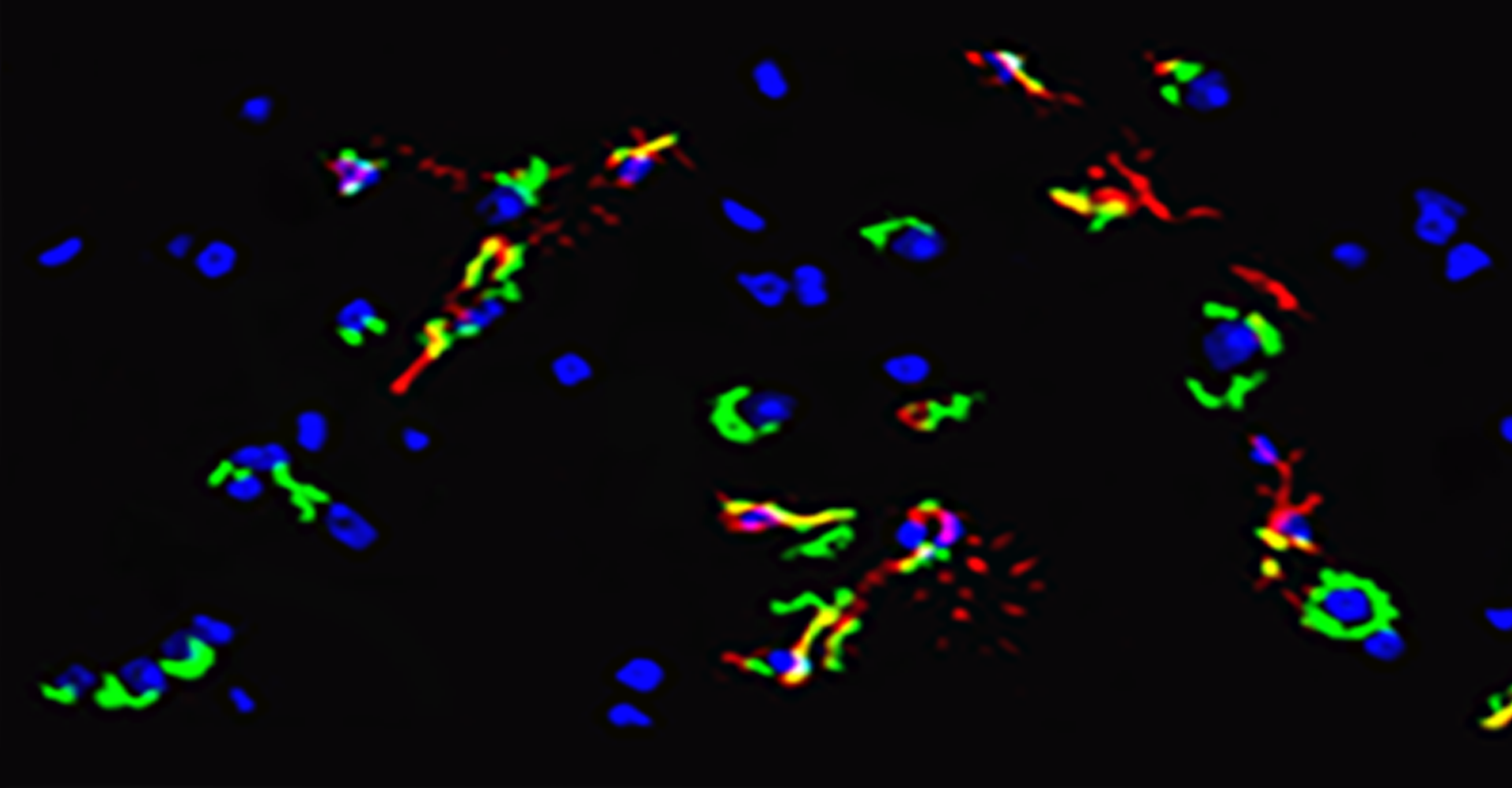

Supplement: Supplementary file 1 [file DataSheet_1.zip › Original Images/Figure 2A GFAP.tif]

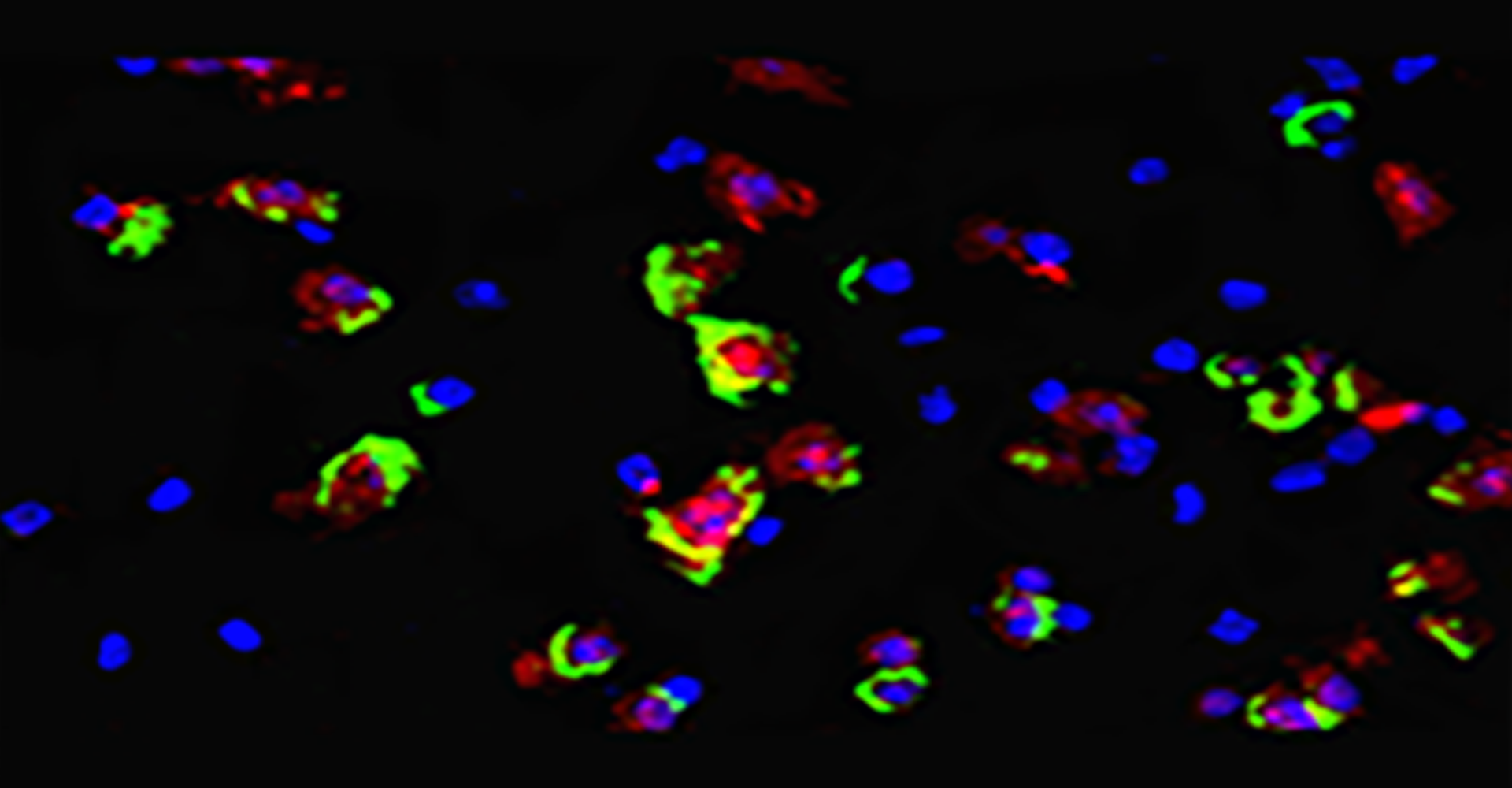

Supplement: Supplementary file 1 [file DataSheet_1.zip › Original Images/Figure 2A Iba-1.tif]

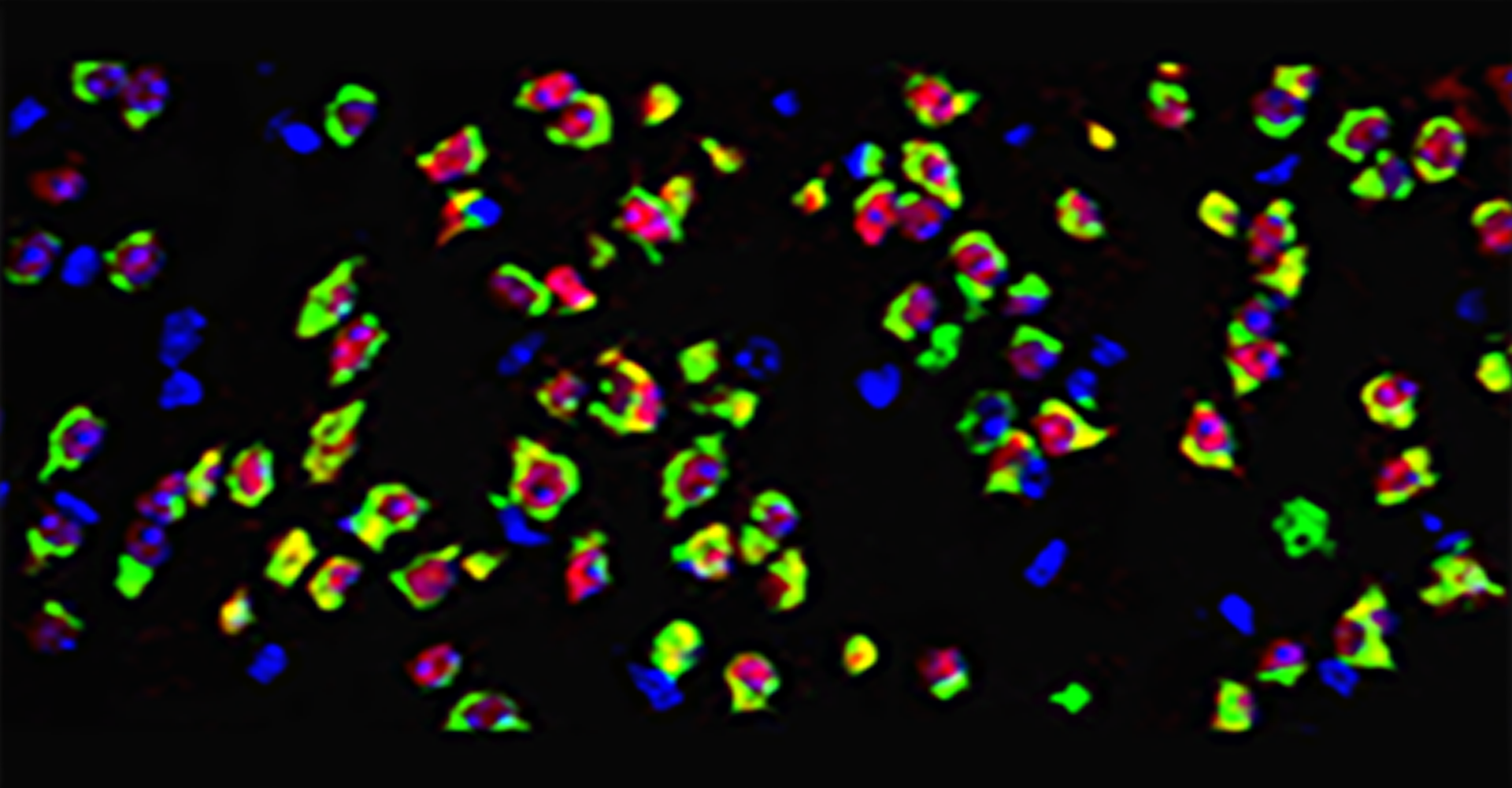

Supplement: Supplementary file 1 [file DataSheet_1.zip › Original Images/Figure 2A NeuN.tif]

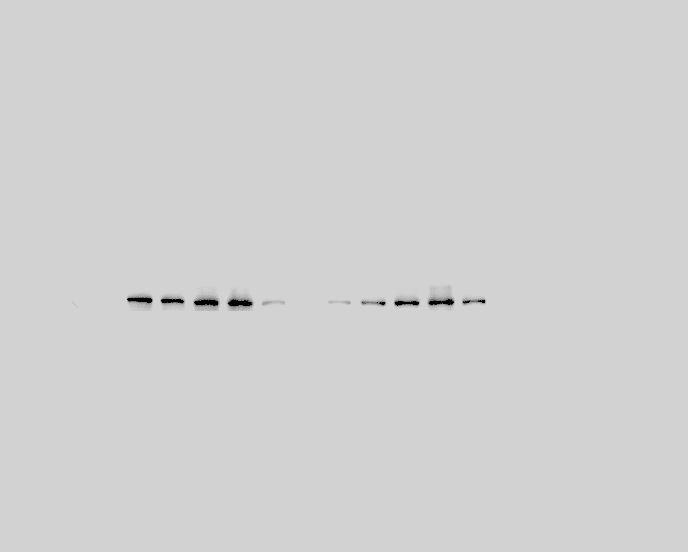

Supplement: Supplementary file 1 [file DataSheet_1.zip › Original Images/Figure 2B A20.tif]

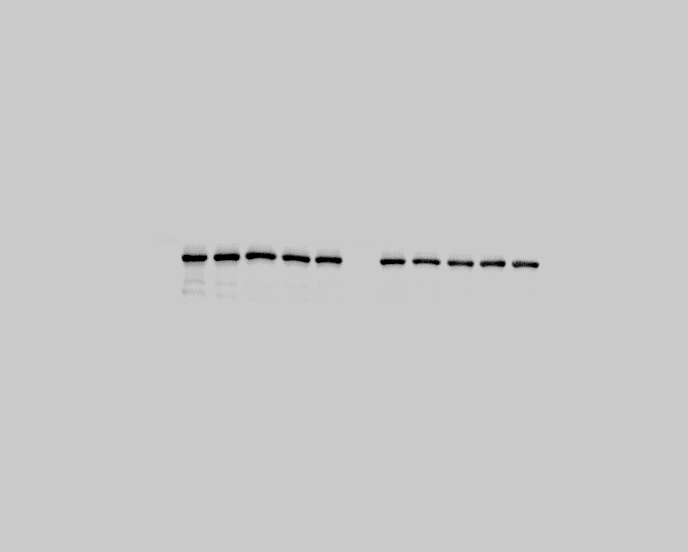

Supplement: Supplementary file 1 [file DataSheet_1.zip › Original Images/Figure 2B β-actin.tif]

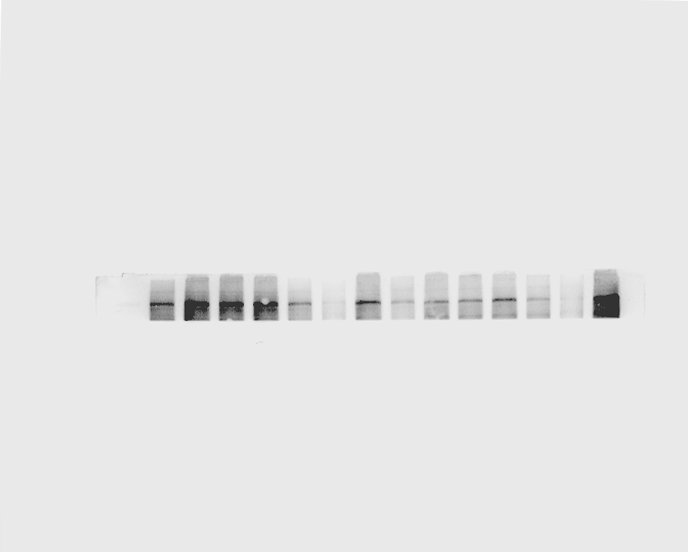

Supplement: Supplementary file 1 [file DataSheet_1.zip › Original Images/Figure 3A A20 .tif]

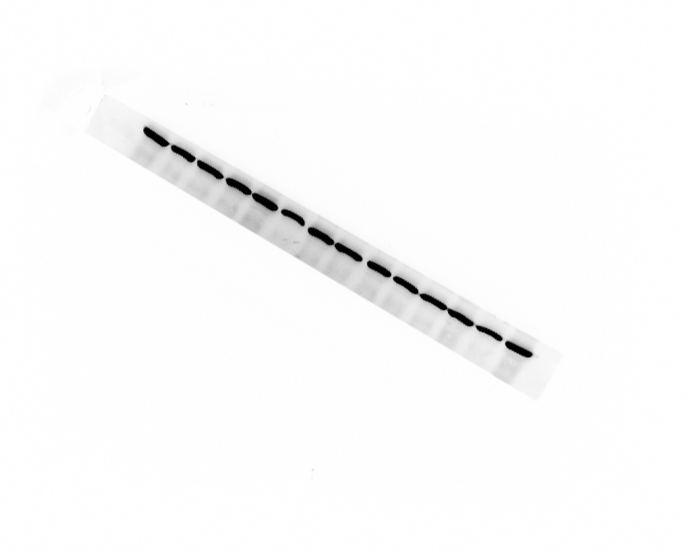

Supplement: Supplementary file 1 [file DataSheet_1.zip › Original Images/Figure 3A&B β-actin.tif]

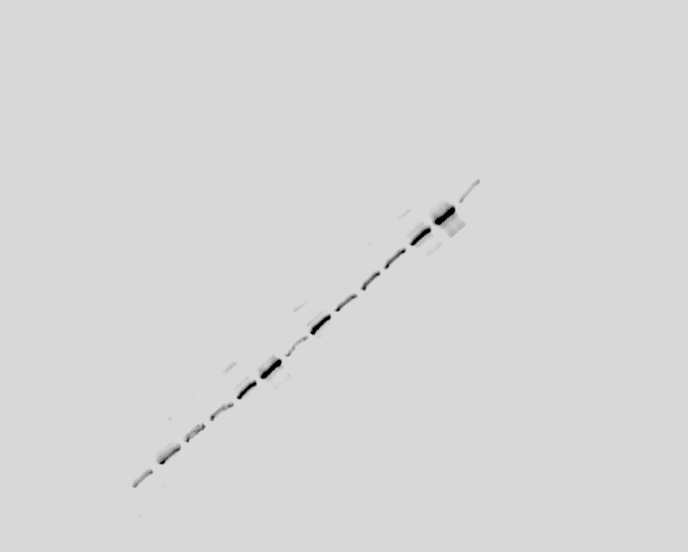

Supplement: Supplementary file 1 [file DataSheet_1.zip › Original Images/Figure 3B TRAF6.tif]

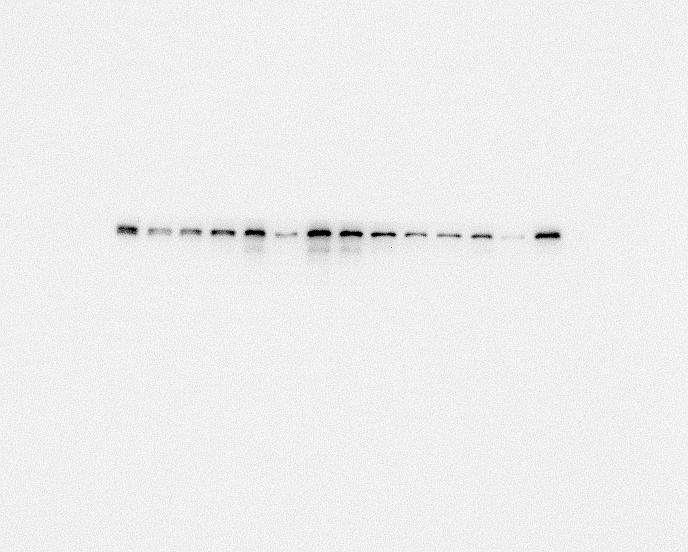

Supplement: Supplementary file 1 [file DataSheet_1.zip › Original Images/Figure 3C IKB-α.tif]

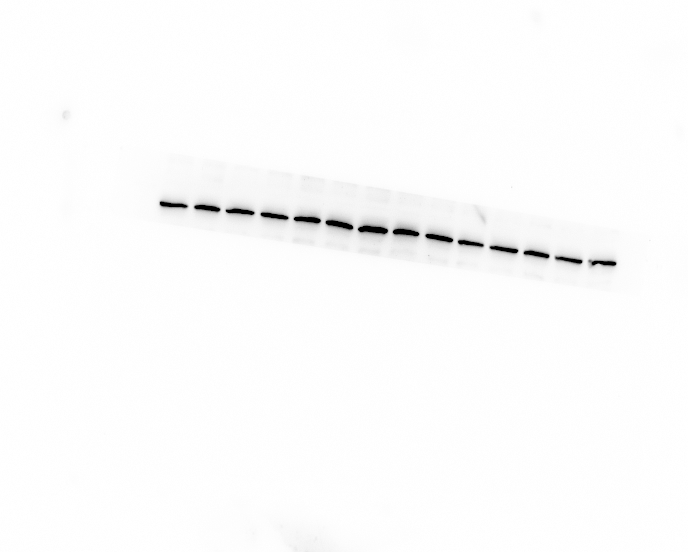

Supplement: Supplementary file 1 [file DataSheet_1.zip › Original Images/Figure 3C&D β-actin.tif]

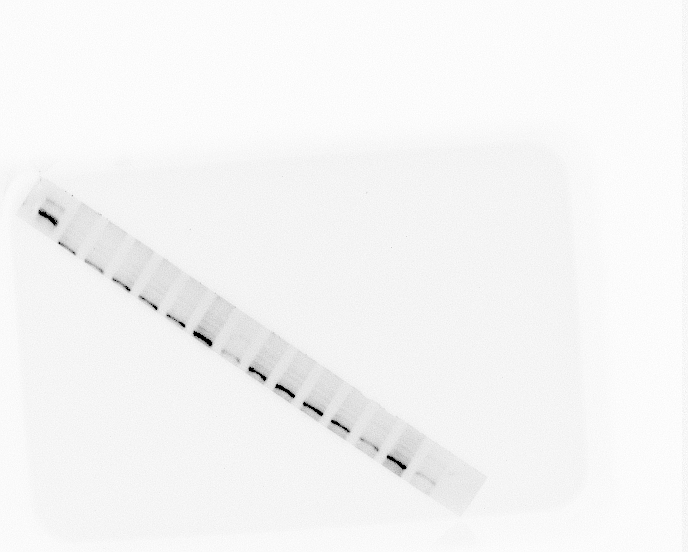

Supplement: Supplementary file 1 [file DataSheet_1.zip › Original Images/Figure 3D P-p65.tif]

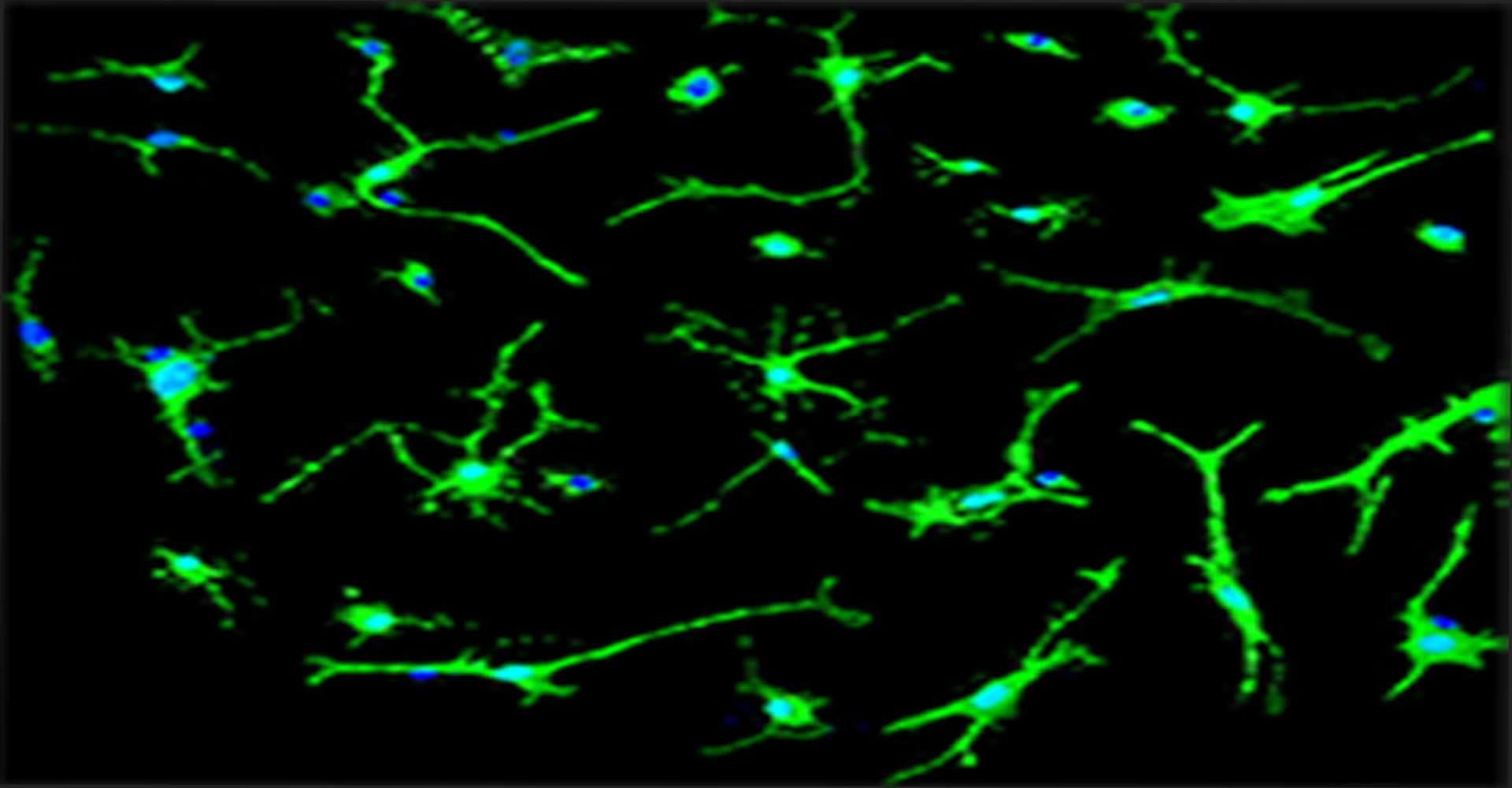

Supplement: Supplementary file 1 [file DataSheet_1.zip › Original Images/Figure 4A.tif]

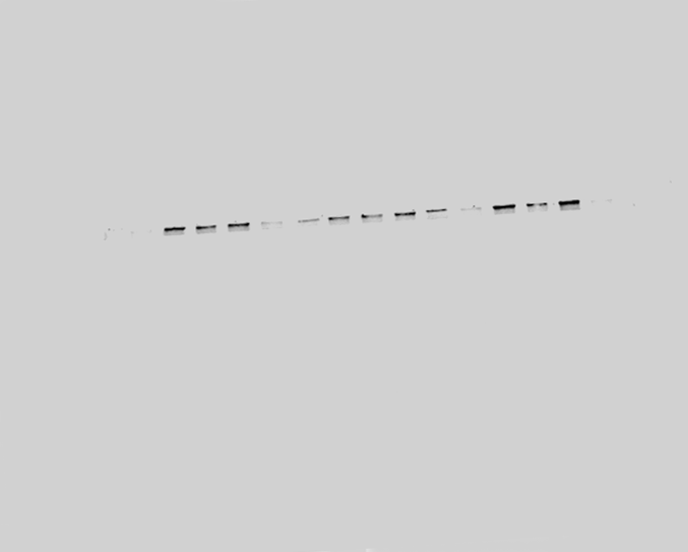

Supplement: Supplementary file 1 [file DataSheet_1.zip › Original Images/Figure 5A MMP-9.tif]

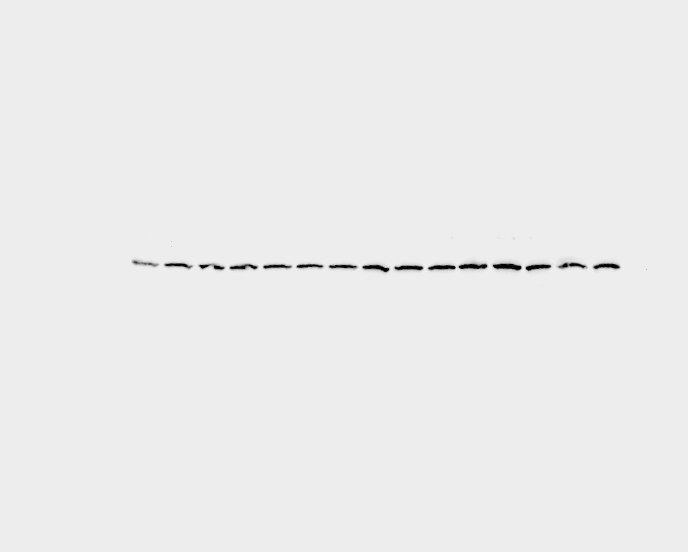

Supplement: Supplementary file 1 [file DataSheet_1.zip › Original Images/Figure 5A&B β-actin.tif]

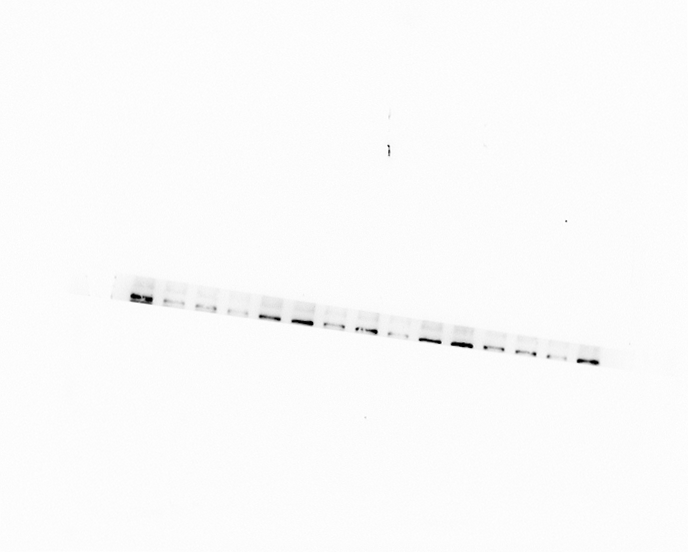

Supplement: Supplementary file 1 [file DataSheet_1.zip › Original Images/Figure 5B ZO-1.tif]

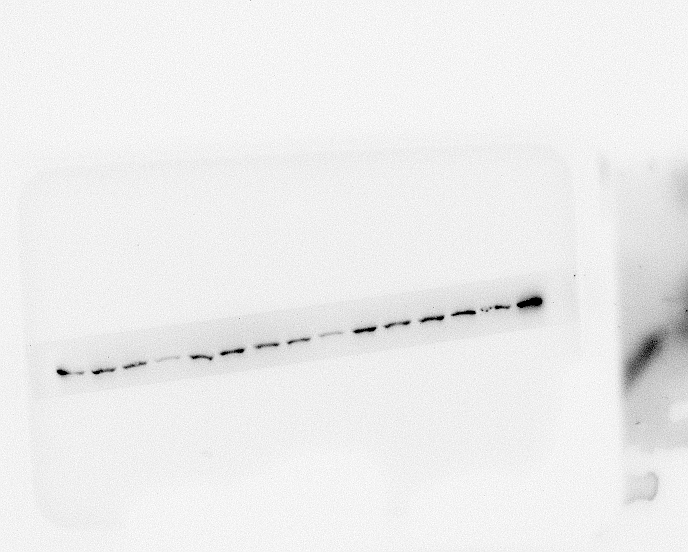

Supplement: Supplementary file 1 [file DataSheet_1.zip › Original Images/Figure 6A Bcl-2.tif]

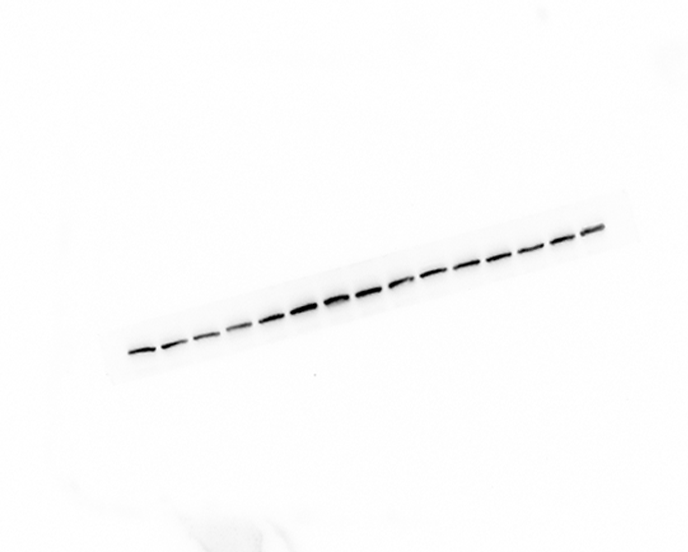

Supplement: Supplementary file 1 [file DataSheet_1.zip › Original Images/Figure 6A&B β-actin.tif]

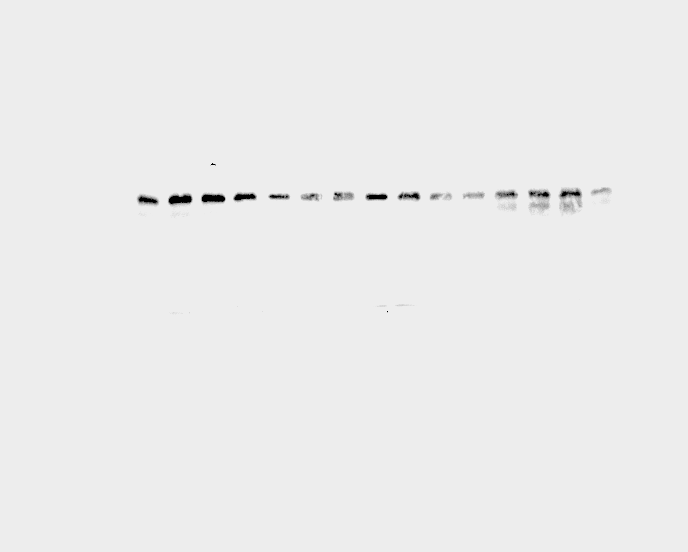

Supplement: Supplementary file 1 [file DataSheet_1.zip › Original Images/Figure 6B Bax.tif]

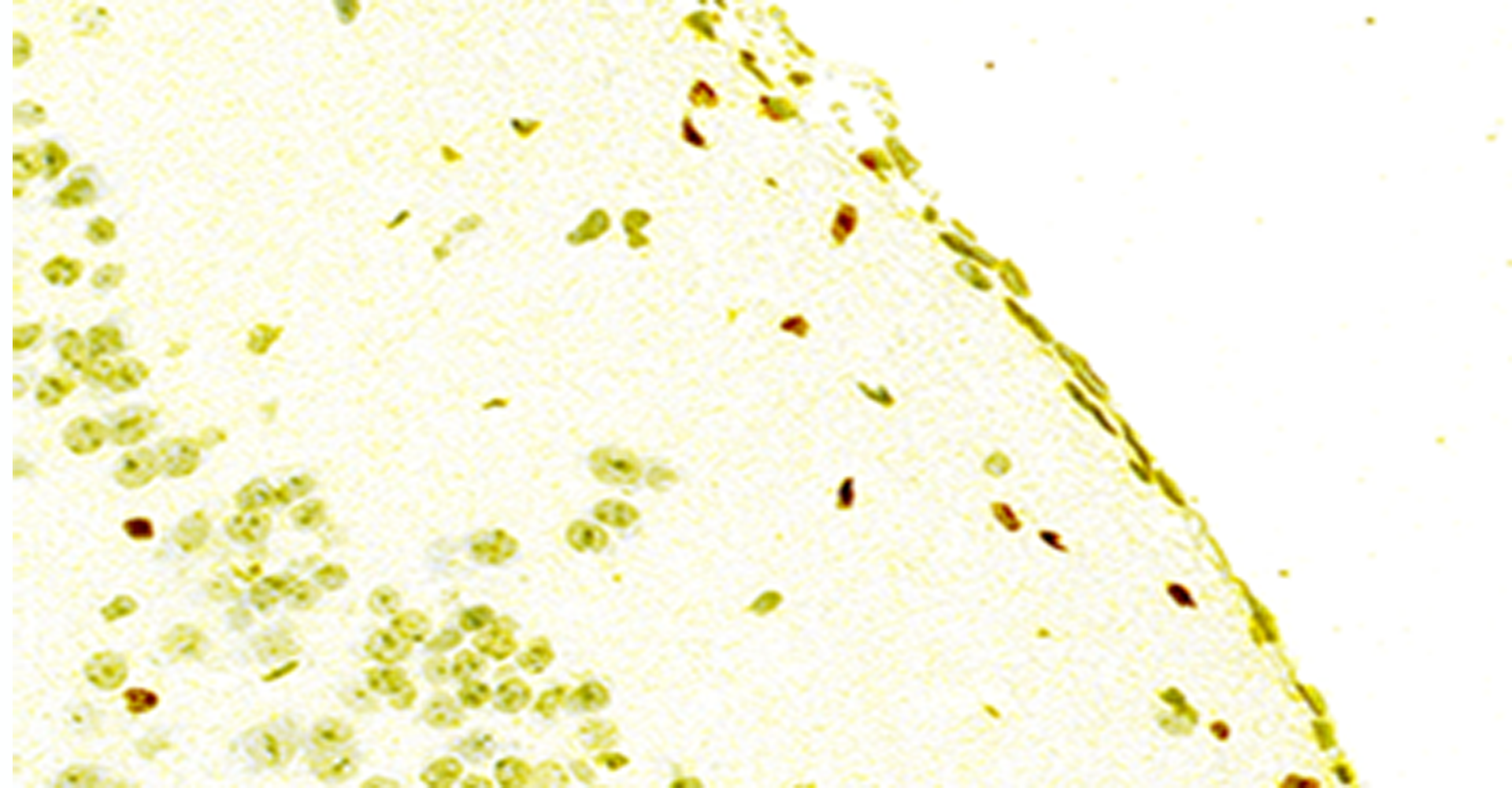

Supplement: Supplementary file 1 [file DataSheet_1.zip › Original Images/Figure 6D SHAM.tif]

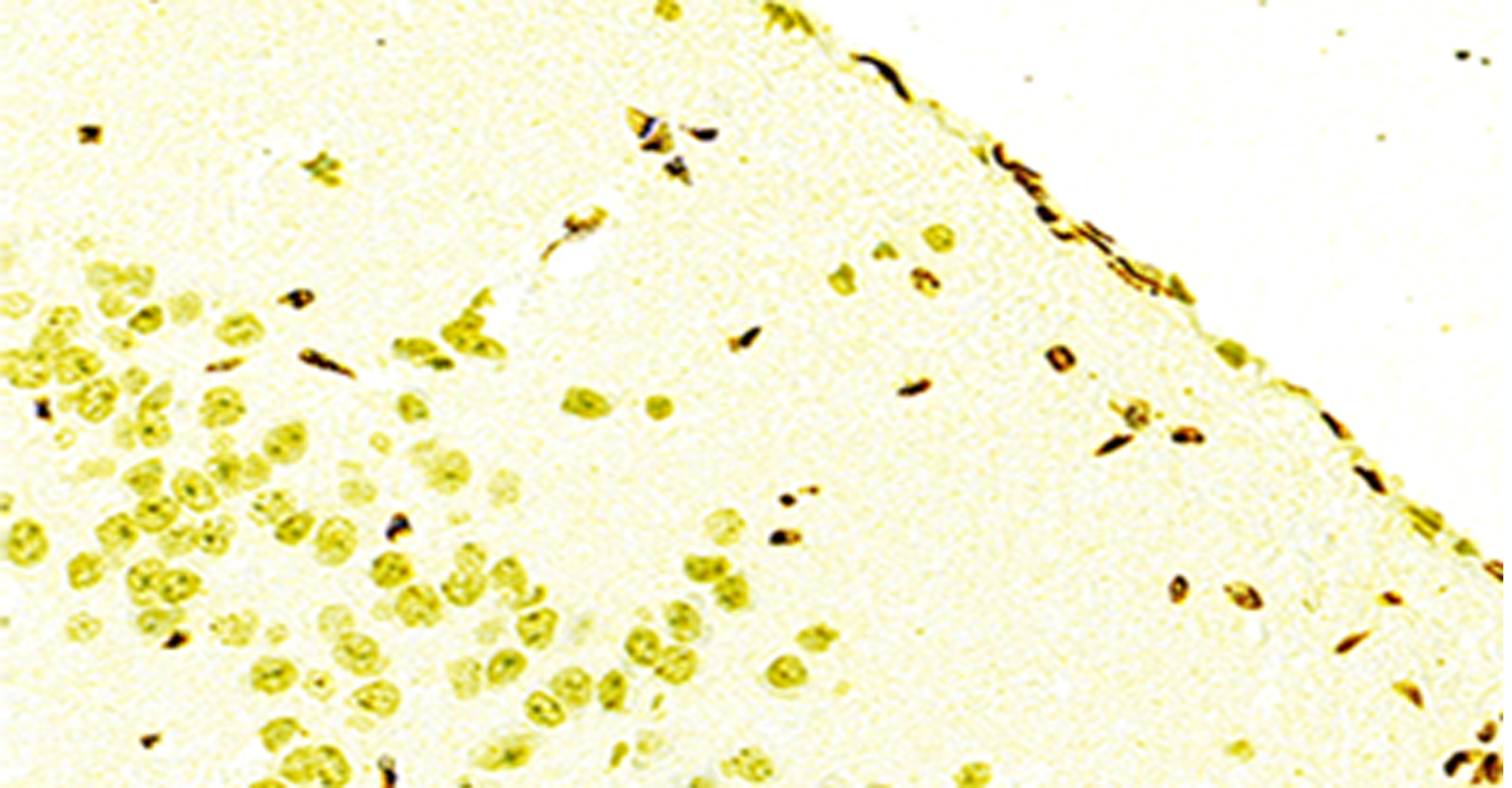

Supplement: Supplementary file 1 [file DataSheet_1.zip › Original Images/Figure 6E SAH.tif]

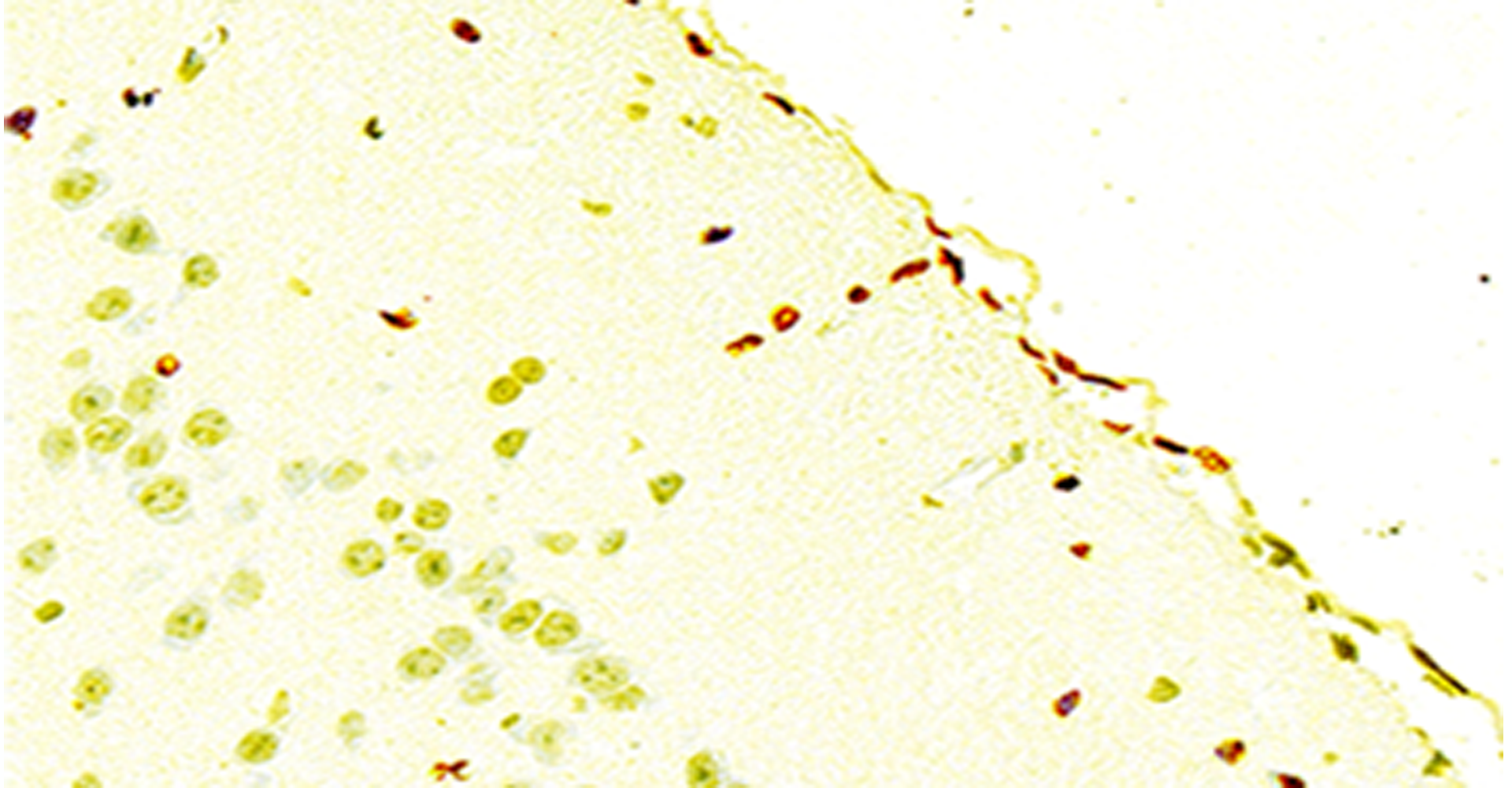

Supplement: Supplementary file 1 [file DataSheet_1.zip › Original Images/Figure 6F SAH+Lv.GFP.tif]

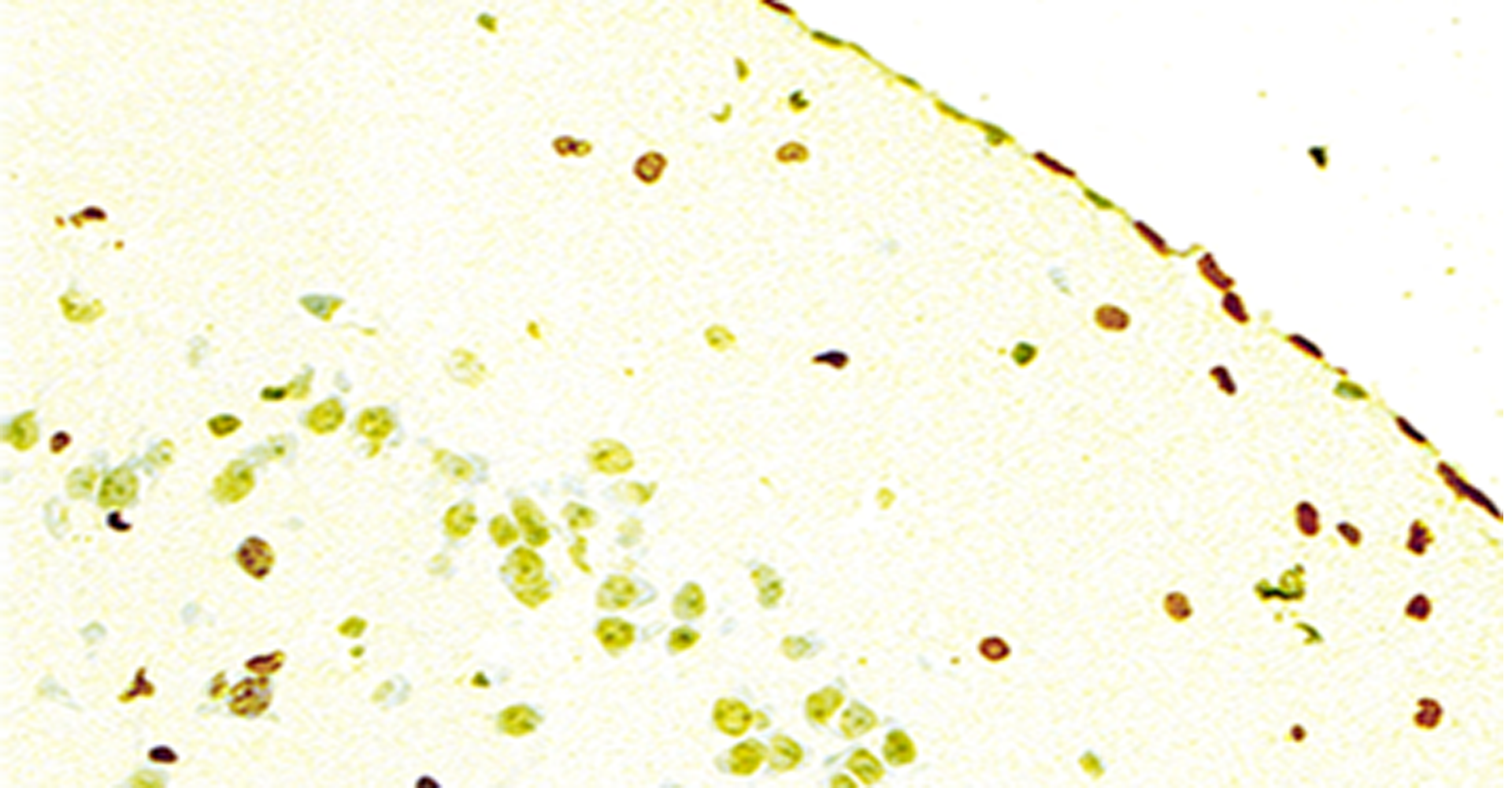

Supplement: Supplementary file 1 [file DataSheet_1.zip › Original Images/Figure 6G SAH+Lv.A20(-).tif]

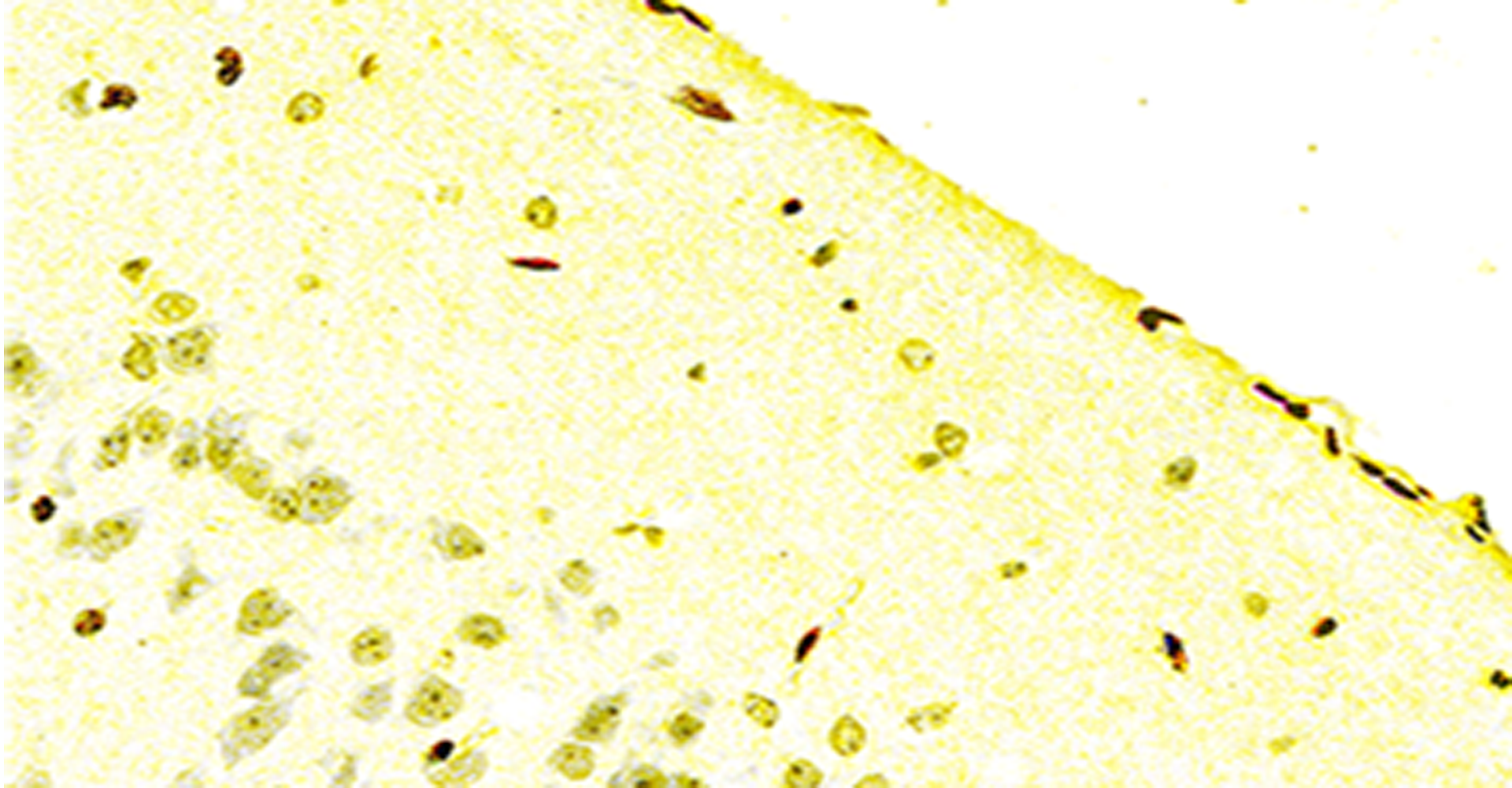

Supplement: Supplementary file 1 [file DataSheet_1.zip › Original Images/Figure 6H SAH+Lv.A20(+).tif]

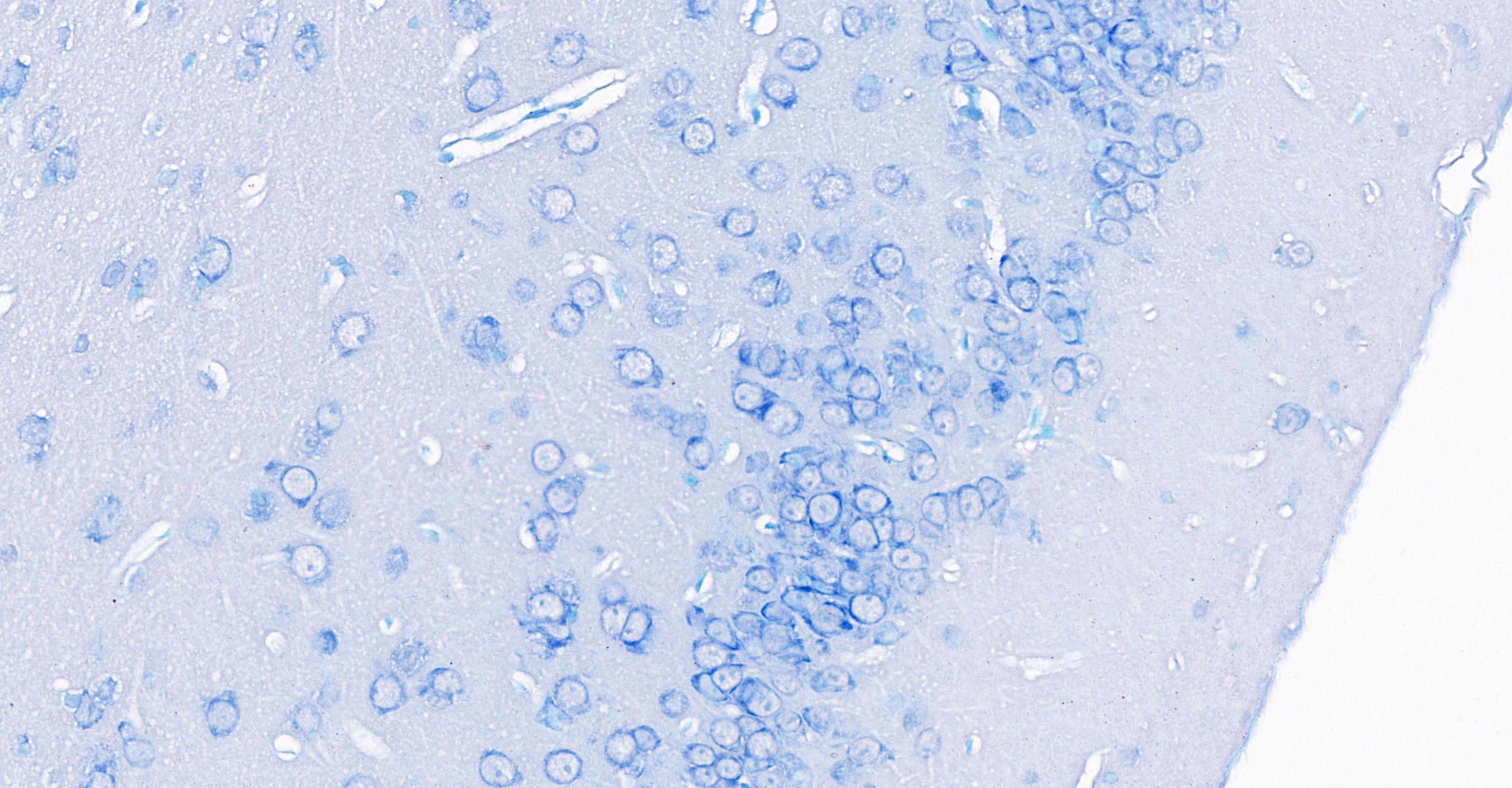

Supplement: Supplementary file 1 [file DataSheet_1.zip › Original Images/Figure 7B SHAM.jpg]

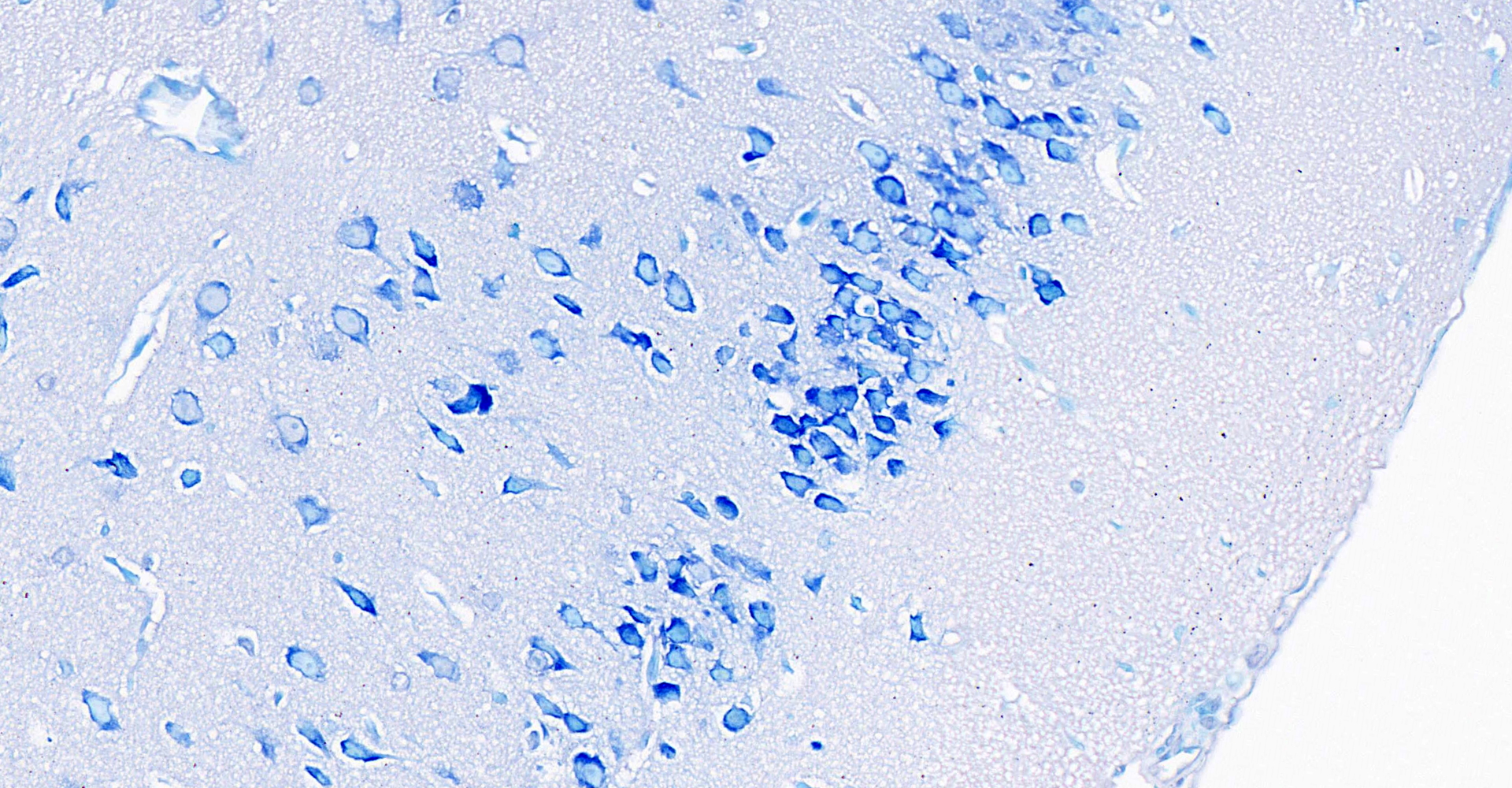

Supplement: Supplementary file 1 [file DataSheet_1.zip › Original Images/Figure 7C SAH.jpg]

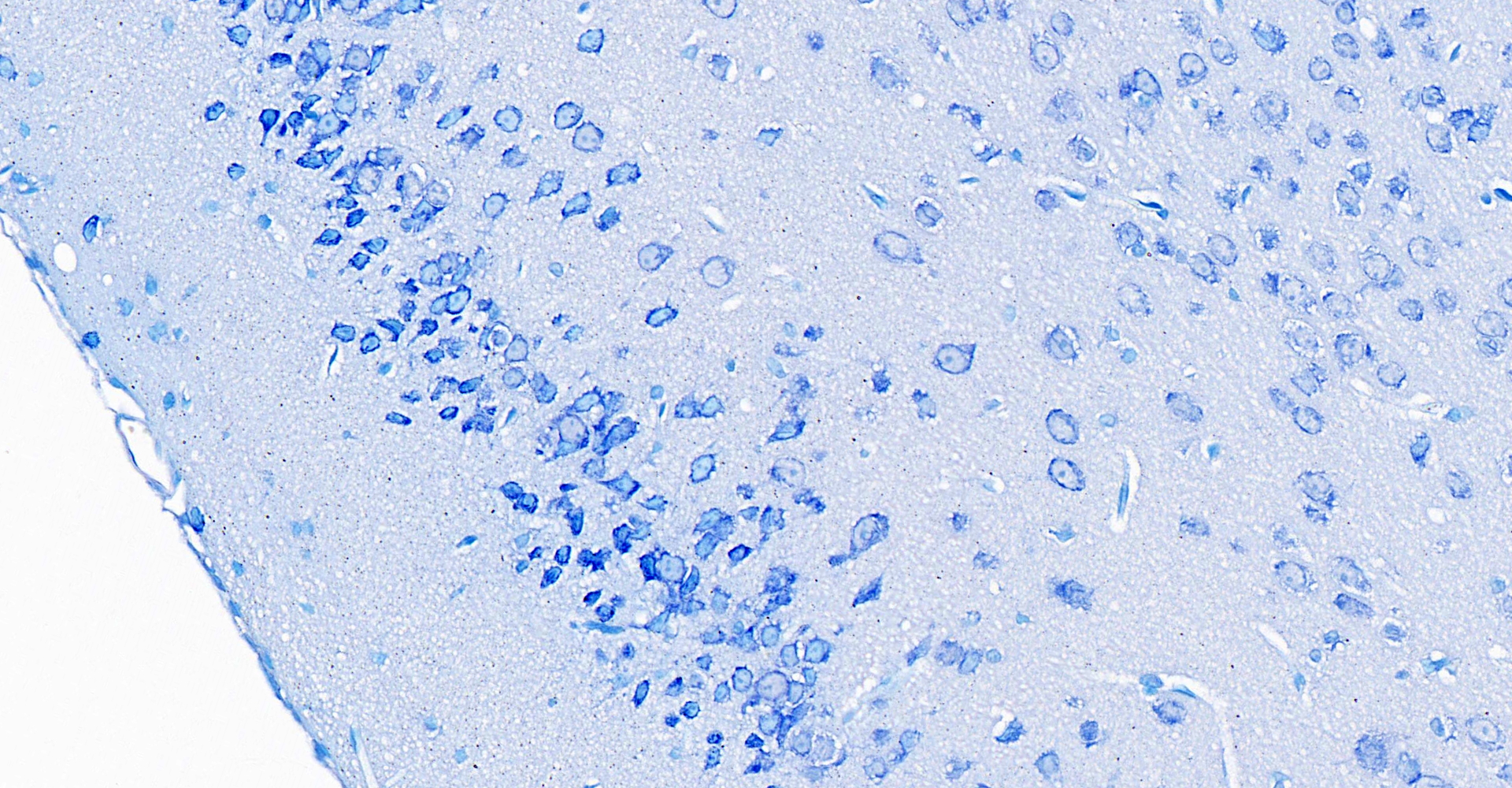

Supplement: Supplementary file 1 [file DataSheet_1.zip › Original Images/Figure 7D SAH+Lv.GFP.jpg]

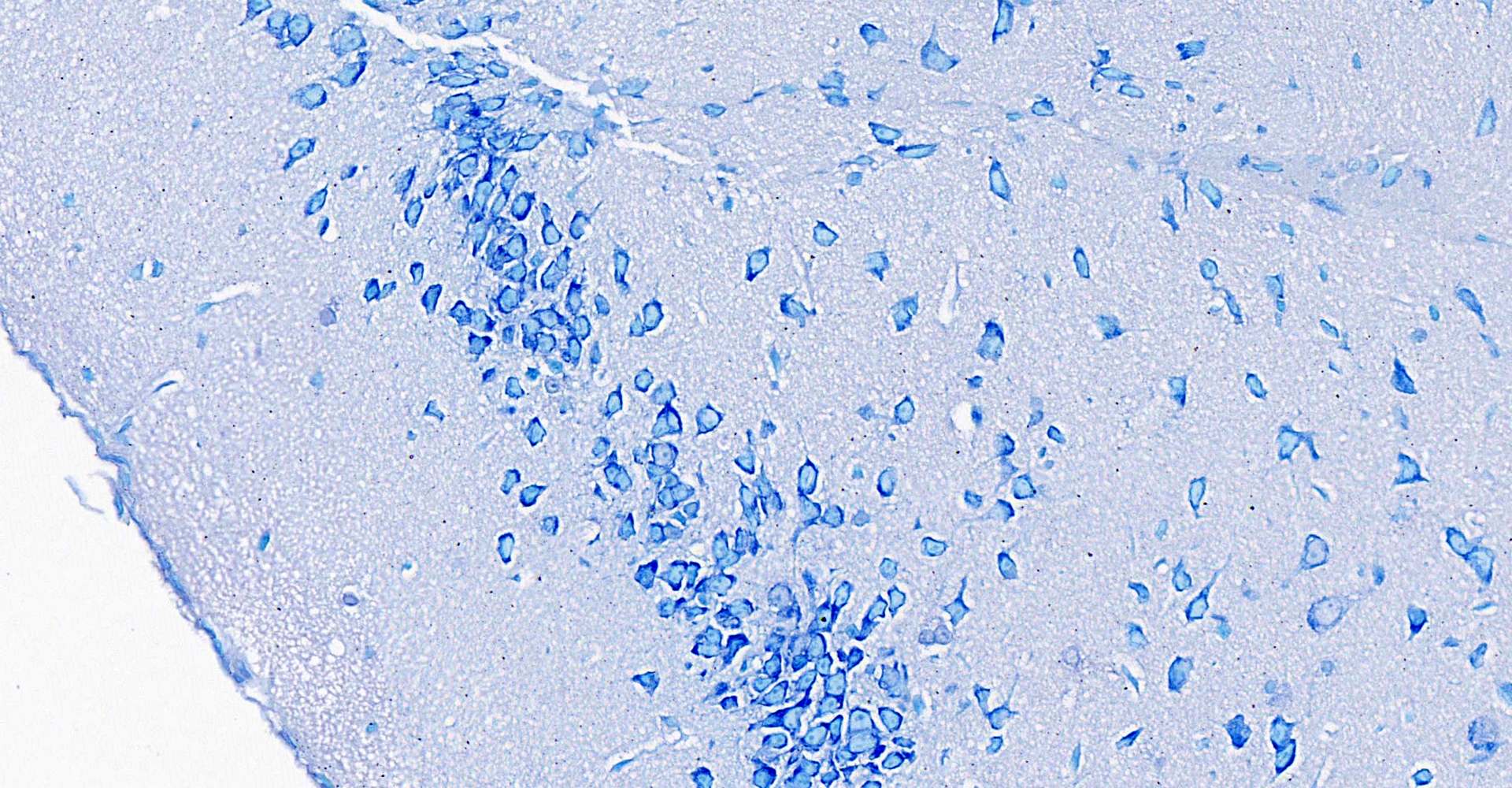

Supplement: Supplementary file 1 [file DataSheet_1.zip › Original Images/Figure 7E SAH+Lv.A20(-).jpg]

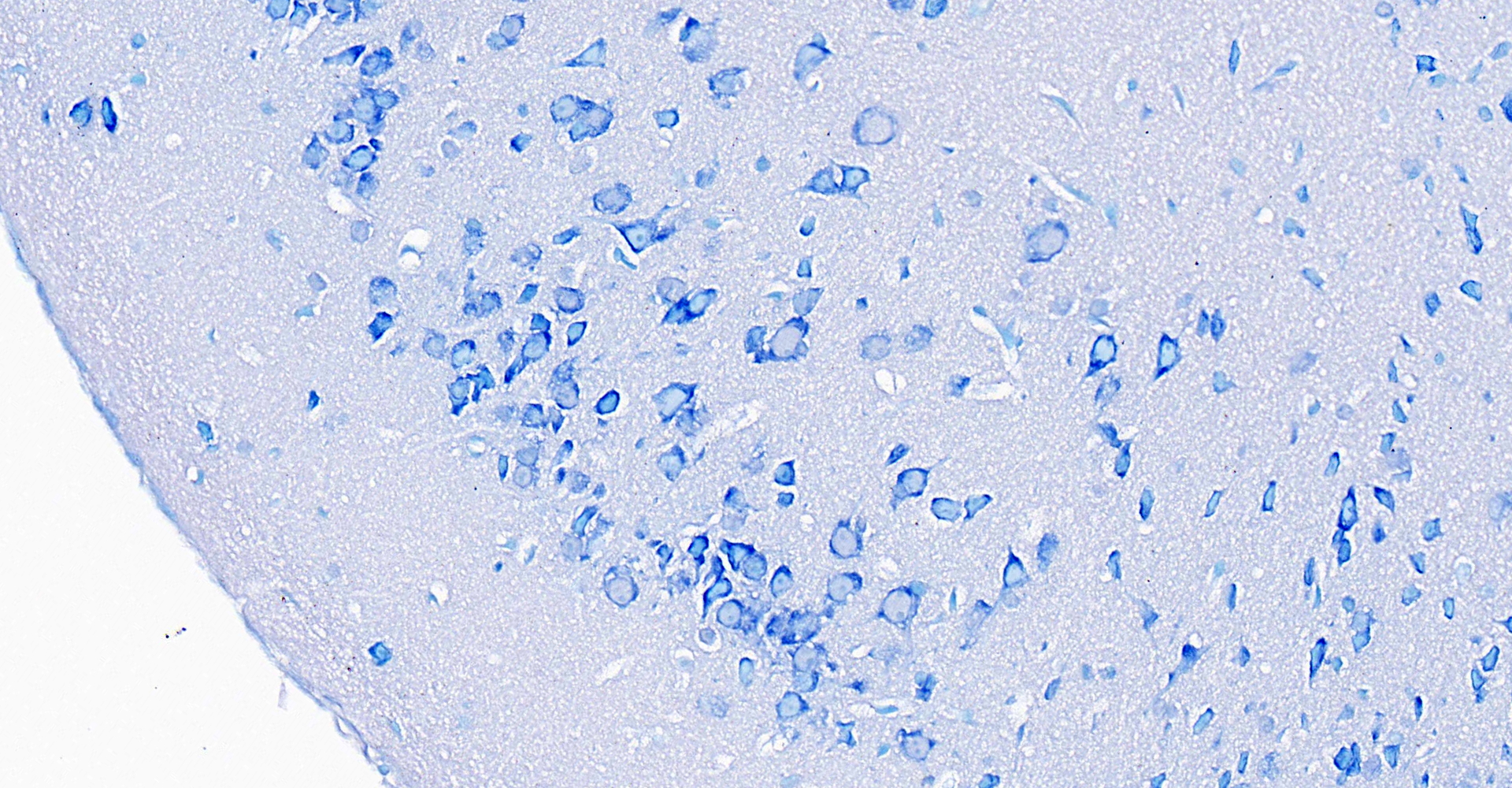

Supplement: Supplementary file 1 [file DataSheet_1.zip › Original Images/Figure 7F SAH+Lv.A20(+).jpg]
